# Mitochondrial perturbations in low-protein-diet-fed mice are associated with altered neutrophil development and effector functions

**DOI:** 10.1016/j.celrep.2024.114493

**Published:** 2024-07-18

**Authors:** Mehakpreet K. Thind, Emiliano Miraglia, Catriona Ling, Meraj A. Khan, Aida Glembocki, Celine Bourdon, YueYing ChenMi, Nades Palaniyar, Michael Glogauer, Robert H.J. Bandsma, Amber Farooqui

**Affiliations:** 1 Department of Nutritional Sciences, Faculty of Medicine, University of Toronto, Toronto, ON, Canada; 2 Translational Medicine Program, The Hospital for Sick Children, Toronto, ON, Canada; 3 The Childhood Acute Illness & Nutrition Network (CHAIN), Nairobi, Kenya; 4 Department of Biochemistry, University of Toronto, Toronto, ON, Canada; 5 Cell Biology Program, Hospital for Sick Children, Toronto, ON, Canada; 6 Laboratory Medicine and Pathobiology, Faculty of Medicine, University of Toronto, Toronto, ON, Canada; 7 Division of Pathology, The Hospital for Sick Children, Toronto, ON, Canada; 8 Institute of Medical Sciences, Faculty of Medicine, University of Toronto, Toronto, Canada; 9 Faculty of Dentistry, University of Toronto, Toronto, ON, Canada; 10 Department of Dental Oncology and Maxillofacial Prosthetics, Princess Margaret Cancer Centre, University Health Network, Toronto, ON, Canada

**Keywords:** immunometabolism, neutrophils, malnutrition, low-protein diet, cellular metabolism, development and functions

## Abstract

Severe malnutrition is associated with infections, namely lower respiratory tract infections (LRTIs), diarrhea, and sepsis, and underlies the high risk of morbidity and mortality in children under 5 years of age. Dysregulations in neutrophil responses in the acute phase of infection are speculated to underlie these severe adverse outcomes; however, very little is known about their biology in this context. Here, in a lipopolysaccharide-challenged low-protein diet (LPD) mouse model, as a model of malnutrition, we show that protein deficiency disrupts neutrophil mitochondrial dynamics and ATP generation to obstruct the neutrophil differentiation cascade. This promotes the accumulation of atypical immature neutrophils that are incapable of optimal antimicrobial response and, in turn, exacerbate systemic pathogen spread and the permeability of the alveolocapillary membrane with the resultant lung damage. Thus, this perturbed response may contribute to higher mortality risk in malnutrition. We also offer a nutritional therapeutic strategy, nicotinamide, to boost neutrophil-mediated immunity in LPD-fed mice.

## Highlights

• Low-protein diet (LPD) exacerbates disease susceptibility in response to inflammatory insults
• LPD increases immature neutrophils and impairs neutrophil functions
• Neutrophil accumulation is dependent on altered neutrophil development and cellular metabolism
• Supplementation with nicotinamide reverses the effect of LPD on neutrophil homeostasis

## Graphical abstract

**Figure:**
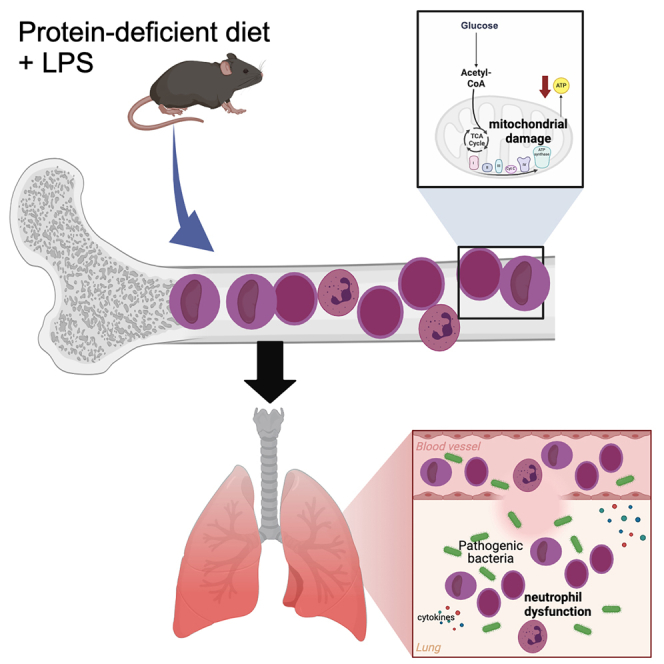

## Teaser

The effect of protein restriction on neutrophil biology for a suboptimal immune response is not well understood. Thind et al. demonstrate that protein deficiency alters neutrophil metabolism, which can influence neutrophil differentiation and functional properties for a disturbed response against microbial products.

## Introduction

Bacterial infections and associated systemic inflammation are major contributors to hospitalization and mortality in acutely ill malnourished children under 5 years of age in mainly Sub-Saharan Africa and South Asia.^1^^,^^2^^,^^3^ The vulnerability to common infections, such as gastroenteritis or pneumonia, in malnourished children underscores the intimate relationship between immunity and nutrition.^3^^,^^4^^,^^5^ It is generally well accepted that malnutrition-associated immunodeficiency contributes largely to increased susceptibility to infection.^6^^,^^7^ Unraveling the mechanisms underpinning this immunodeficiency are therefore needed to identify novel approaches to improve outcomes in childhood malnutrition.

Neutrophils are critical to examine in malnutrition-induced infection risk, as they are the most abundant and indispensable effector innate immune cells that respond rapidly against sterile and microbial insults in areas under inflammatory attack. In the bone marrow (BM), hematopoietic stem cells (HSCs) proliferate and differentiate to give rise to all blood cell lineages. Early myeloid committed precursors give rise to mature neutrophils through stepwise differentiation into stages involving a tight control of gene regulatory networks.^8^^,^^9^^,^^10^^,^^11^^,^^12^ Alterations in core transcription factor (TF) expression, especially in the BM, are known to greatly influence neutrophil numbers and effector properties.^9^^,^^10^^,^^13^^,^^14^^,^^15^ Additionally, mitochondrial fatty acid oxidation (FAO), and oxidative phosphorylation (OXPHOS) provide ATP to enable these signals to drive neutrophil differentiation, where impaired FAO and mitochondrial ATP depletion result in defective neutrophil differentiation marked by an accumulation of immature neutrophils.^16^

Neutrophils’ capacity for chemotaxis, phagocytosis, degranulation, NADPH oxidase-dependent reactive oxygen species (ROS) production, and neutrophil extracellular trap generation allows for the effective containment of pathogens. It is well established that neutrophil development and function are closely linked.^9^^,^^12^^,^^13^^,^^16^^,^^17^ Perturbations in some human blood neutrophil functions have been reported in severe malnutrition including reduction in chemotaxis and bactericidal mechanisms, while reports on the impact of malnutrition on phagocytic capacity have been inconsistent.^5^^,^^6^^,^^7^ The interplay between neutrophil development and function remains unexplored in malnutrition.

It is generally well accepted that immune responses against pathogens are costly in terms of metabolic and energetic demands; thus, host nutritional status can greatly impact immune cell effector functions and infection outcomes.^18^ Malnutrition is associated with altered protein metabolism and decreased circulating levels of essential amino acids.^19^ In addition, metabolic perturbations have been reported in human cohorts and pre-clinical models of malnutrition.^3^^,^^19^^,^^20^ Here, altered mitochondrial homeostasis was related to intestinal barrier and hepatic dysfunction in pre-clinical models of severe malnutrition.^20^^,^^21^^,^^22^^,^^23^ Additionally, systemic disruption in mitochondria-related bioenergetic pathways (specially tricarboxylic acid cycle metabolites and free fatty acids) and systemic inflammation are strongly associated with morality in children with complicated severe malnutrition^3^^,^^24^. However, the relation between neutrophil metabolism and neutrophil development and function remains unexplored in states of malnutrition.

In this study, we exposed mice to a low-protein diet (LPD) as a model of malnutrition to understand the effect on neutrophil development and function during inflammation and the possible role of altered metabolism here. Using this model diet, mice have reduced plasma concentrations of most essential amino acids,^22^^,^^23^ similar to what is commonly observed in children with severe malnutrition.^19^ We show the accumulation of immature neutrophils related to impaired neutrophil functions in mice fed an LPD. We also demonstrate an association between perturbed neutrophil metabolism and malnutrition-induced neutrophil dysfunction. Importantly, ATP depletion through impaired mitochondrial respiration was associated with limited neutrophil differentiation and accumulation of these immature subsets. Supplementing with nicotinamide (NAM) improved mitochondrial dysfunction and overall neutrophil differentiation.

## Results

### Severe protein restriction enhances susceptibility to pathological inflammation and mortality in mice

**Figure 1:**
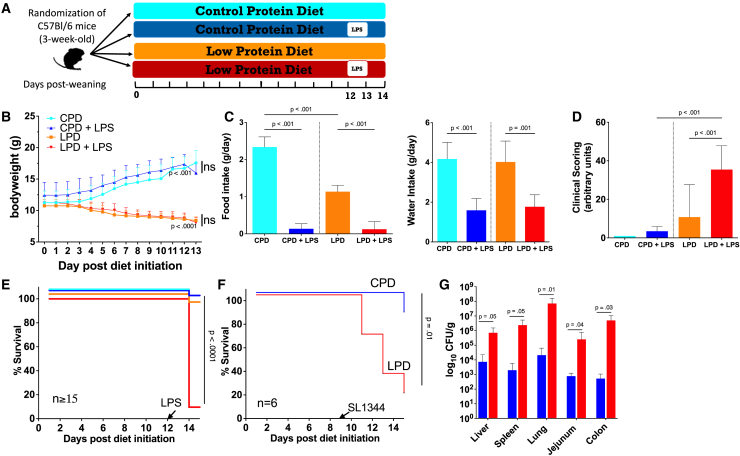
Mice fed an LPD are more susceptible to disease and mortality following endotoxin and live bacteria administration (A) Experimental strategy; weanling C57BL/6 male mice fed control protein diet (CPD; cyan) or low-protein diet (LPD; orange) were subjected to single 4 mg/kg intraperitoneal (i.p.) LPS (B55:05) challenge (CPD+LPS, blue; LPD+LPS, red) and sacrificed 24 h later. (B and C) Bodyweight (*n* = 46/group) (B) and food and water intake (C) are shown following LPS challenge. (D) Clinical severity scoring assessed prior to animal sacrifice (*n* = 13/group). A score of 35 or higher was considered the humane endpoint, and mice were then euthanized. (E) Survival of CPD- and LPD-fed mice during the 2-week experimental period with or without LPS challenge (*n* = 15/group). (F) Survival of CPD- and LPD-fed mice (*n* = 6) orally infected with *S.* Typhimurium (SL1344). (G) Bacterial loads (*n* = 4) of *S.* Typhimurium in orally infected mice on day 2 post-infection in spleen, liver, lung, jejunum, and colon. Survival curves (F and G) include animals that reached the CSS (>35) for humane endpoint or died during the experimental period. Results are expressed as means ± SD as determined by (B) two-way ANOVA and unpaired two-tailed t test analysis, (C and D) one-way ANOVA with Tukey’s multiple comparisons, (E and F) two-sided log rank (Mantel-Cox), or (G) one-way ANOVA.

Severe protein deficiency, as reported before, was used to induce malnutrition.^20^^,^^22^^,^^23^^,^^25^ Weanling C57BL/6 mice fed an LPD, with caloric density and fat content equal to that of the control protein diet (CPD), became underweight (Figure S1A), wasted (Figure S1B), and developed a stunted phenotype (Figures S1C and S1D) compared to the CPD-fed mice with no lethality over the 14-day experimental period. This reflects what has already been observed with other models of feeding LPDs to post-weaned mice.^26^^,^^27^ Protein restriction led to higher water intake and lower absolute food intake over the experimental period, but no difference was observed in food intake normalized to bodyweight (Figures S1E and S1F). Using this model diet, we first aimed to recapitulate acute systemic inflammation through a single intraperitoneal injection of endotoxin lipopolysaccharide (LPS *E. coli*; 055: B5) administered on day 12 to CPD- and LPD-fed mice. For dose determination, body weight and mortality were assessed for 48 h post-LPS for all administered doses (Figure 1A). For subsequent experiments, animals were sacrificed 24 h post-LPS treatment since most animals died in the LPD group by 48 h. Significant weight loss was observed in CPD- and LPD-fed mice at 24 h (Figure 1B), consistent with lower food and water intake after LPS challenge (Figure 1C). A higher clinical severity score, as a measure of impaired health status (Figure 1D) and mortality, was found in the LPD-fed compared to the CPD-fed mice (Figure 1E). However, no mice in the LPD or CPD groups died or reached the CSS threshold within 24 h post-LPS and, therefore, were not excluded from further analysis. A dose-dependent response of LPS was observed in LPD mice administrated a single intraperitoneal injection of varying doses of endotoxin LPS (LPS *E. coli*; 055: B5) (Figure S2). In line with the LPS findings, oral infection with *Salmonella* Typhimurium (SL1344), a common gram-negative bacterium for mortality in children with infection and severe malnutrition, also led to higher mortality, intestinal bacterial growth, and systemic spread of viable bacteria into the liver, spleen, and lungs (Figures 1F and 1G). Overall, these data indicated that LPD-fed mice are more susceptible to disease and mortality following LPS or live gram-negative bacteria challenge.

### LPD aggravates neutrophilic inflammation following LPS challenge

**Figure 2:**
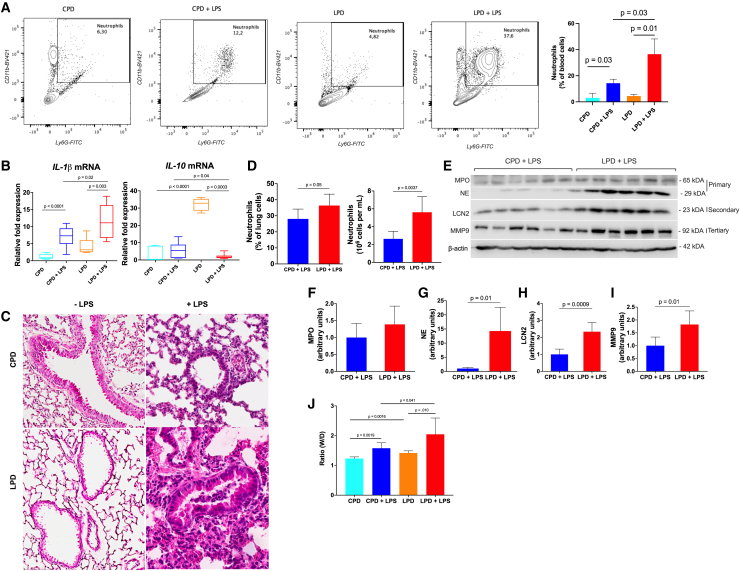
Severe protein restriction aggravates neutrophilic inflammation following LPS challenge (A) Representative gating strategy (left) of blood cells to identify neutrophils. Numerical values represent the percentage of cells within each gate. The percentage (right) of neutrophils (Ly6G^+^CD11b^+^ cells) in the blood (*n* = 6/group) is shown. (B) Box and whisker plots of mRNA expression of cytokines (normalized to expression of Actin b in CPD). (C) Histological features of mouse lung (H&E, 20×). Scale bar, 200 μm. (D) The percentage (left) and absolute number (right) of neutrophils (Ly6G^+^ cells) in the lungs (*n* = 6/group). (E) Representative western blot probed for MPO, NE, LCN-2, and MMP9 and β-actin in lung lysates is shown. (F–I) Quantification of (F) MPO, (G) NE, (H) LCN-2, and (I) MMP9 normalized to the amount of β-actin was calculated (*n* = 6/group). (J) Lung damage was evaluated by the lung wet/dry (W/D) ratio. Results are expressed as means ± SD as determined by (A, B, and J) one-way ANOVA with Tukey’s multiple comparisons test or (D and F–I) unpaired two-tailed t test analysis.

To further determine whether the higher susceptibility to LPS in LPD-fed mice was related to an exaggerated inflammatory phenotype, we assessed cellular infiltration in the circulation and tissues, as neutrophil numbers are implicated in inflammatory disease states. Through flow cytometry, we found a higher percentage of neutrophils in the systemic circulation in LPS-challenged LPD-fed mice compared to CPD-fed mice (Figure 2A). Consistent with bacterial spread in the lungs (Figure 1G), the pro-inflammatory cytokine interleukin (IL)-1b was markedly higher, while anti-inflammatory cytokine IL-10 mRNA expression was lower, in LPD-fed mice lung lysates compared to lysates from CPD-fed mice in response to LPS challenge (Figure 2B; Figure S3A). Protein restriction alone did not impact the inflammatory status in the mice (Figures S3B–S3F) but increased pulmonary inflammation as evaluated through histology of the lungs, multiparametric flow cytometry, and western blot of inflammatory proteins in the LPS-challenged mice. This LPS-induced inflammation may be driven mostly by neutrophils, where neutrophil count and percentage as well neutrophil-specific proteins (NE, LCN-2, and MMP9) were higher (Figures 2C–2I). Similarly, the ratio of wet lung to dry lung (W/D ratio), as an indicator of lung injury with pulmonary permeability and edema, was higher in LPD-fed mice upon LPS challenge (Figure 2J). No difference was found in neutrophil count and percentage in the peritoneal exudates (Figures S3G and S3H). Overall, these data suggested that LPD-fed mice are more susceptible to LPS-induced neutrophil-tissue infiltration and inflammation.

### LPD feeding leads to an accumulation of dysfunctional neutrophils and exacerbates inflammation

**Figure 3:**
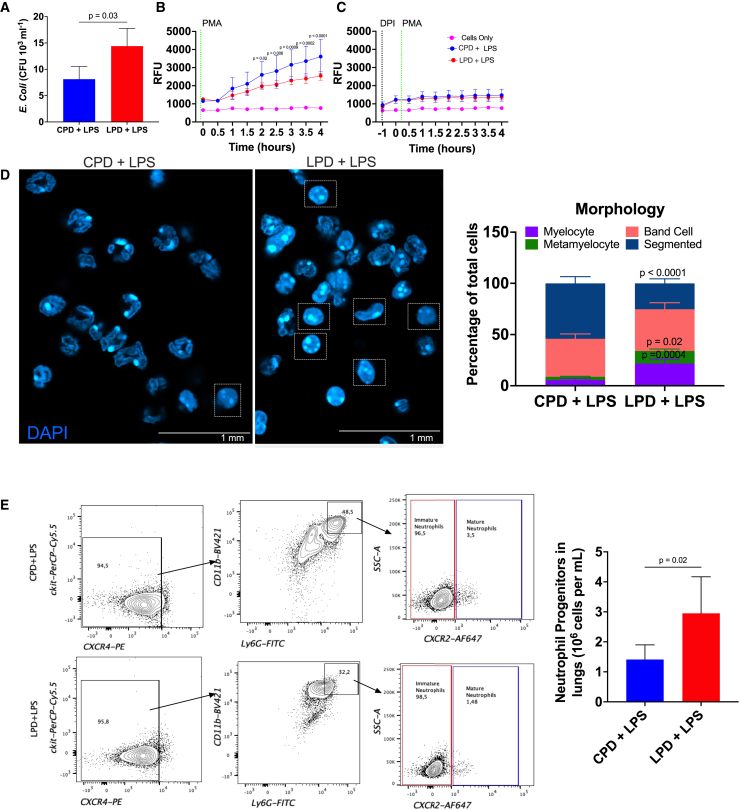
Severe protein restriction alters neutrophils’ antimicrobial functions (A) *In vitro* bacterial killing with CD11b^+^Ly6G^+^ lung neutrophils incubated with *E. coli* at multiplicity of infection (MOI) of 10 for 3 h before cell lysis (*n* = 3/group). Live intracellular bacteria that escaped killing were measured on agar plates and plotted as absolute numbers of colony-forming units (CFUs) × 10^3^ per mL. (B and C) Comparison of ROS production in magnetically sorted lung neutrophils from LPS-challenged C57BL/6 mice. Purified neutrophils (CD11b^+^Ly6G^+^) were incubated with (B) only 10 μM of cell-permeable DHR123 and stimulated with 50 nM PMA (with stimulation) (*n* = 9/group) or (C) 10 μM of cell-permeable DHR123 and 2.5 μM of NADPH oxidase inhibitor DPI (*n* = 6/group) for 1 h at 37°C and stimulated with 50 nM PMA (with inhibitor and stimulation). Each sample was analyzed in triplicate wells. Results are expressed as means ± SD of two independent experiments; ^∗^*p* < 0.05, ^∗∗^*p* < 0.01, ^∗∗∗^*p* < 0.001, and ^∗∗∗∗^*p* < 0.0001; NS, not significant as determined by two-way ANOVA with Tukey’s multiple comparisons test. The black dotted line in (C) shows when 2.5 μM DPI was added, and the green dotted lines in (B) and (C) show when cells were stimulated with 50 nM PMA. (D) Representative confocal microscopy images of morphology assessment of CPD and LPD lung neutrophils (left). Images were obtained under a microscope with a 63× objective. Scale bar, 1 mm. At least 100 cells were counted from different fields from (D), and different maturation stages in CPD and LPD lung neutrophils were quantified (*n* = 3/group). The results are expressed as percentages of myelocytes, metamyelocytes, and band and segmented neutrophils (right). (E) Gating strategy used to quantify neutrophils under different maturation stages (left). Absolute counts (right) of neutrophil progenitor numbers (ckit^−^CXCR4^−^CD11b^+^Ly6G^+^CXCR2^−^) in the lungs (*n* = 6/group). Results are expressed as means ± SD as determined by (A and E) unpaired two-tailed t test analysis or (D) two-way ANOVA with Šidák’s multiple comparisons test.

We next investigated neutrophil functions in our inflammation model. We focused on lung neutrophils since higher bacterial burden and pathology in the lungs was observed in the LPD-fed mice. In line with the higher *in vivo* systemic bacterial load, LPD affected the ability of neutrophils to kill bacteria *in vitro*, with higher intracellular survival of *E. coli* (Figure 3A). We observed a significant reduction for *in vitro* ROS production in lung Ly6G^+^ cells stimulated with PMA, a stimulator of protein kinase C activity and, subsequently, NADPH oxidase, in LPD-fed mice compared to CPD-fed mice (Figure 3B). Both NADPH oxidase and mitochondrial-mediated ROS contribute to the intracellular antibacterial defense capability of neutrophils.^12^^,^^28^ To confirm that NADPH oxidase was activated and altered under the conditions used, we used diphenyleneiodonium chloride (DPI), a specific inhibitor of NADPH oxidase function.^29^ We found similar reductions in ROS levels in all groups to levels comparable to unstimulated neutrophils (Figure 3C). Thus, we demonstrate that with DPI and PMA stimulation, differences in ROS production between LPD and CPD lung neutrophils are NADPH oxidase dependent and not mitochondrial. LPD neutrophils have altered NADPH-oxidase-dependent ROS production.

It has been reported that the cellular composition of neutrophils in inflamed tissues affects neutrophil functionality and influences disease outcomes.^9^^,^^13^^,^^16^^,^^30^ We therefore characterized neutrophil cellular composition in the lungs based on nuclear morphology and surface marker expression, as defined in published literature,^12^^,^^31^^,^^32^ that could be associated with altered responses. We found more myeloblast and metamyelocyte-like neutrophils with a rounded and/or kidney-shaped morphology in LPD-fed mice, indicative of an early neutrophil precursor (Figure 3D). Similarly, we found a higher number of progenitors, ckit^−^CXCR4^−^Ly6G^+^CD11b^+^CXCR2^−^ cells, through flow cytometry (Figure 3E). Along these lines, CXCL1 (Figure S4A), a potent neutrophil chemoattractant, levels were unaffected in the lungs of LPD-fed mice. Additionally, a comparison of lung TF expression between CPD-fed and LPD-fed mice showed that there was no difference in the expression of TFs, Junb, and Relb, which are known for tissue-specific regulation of neutrophil effector functions (Figures S4B and S4C).^13^ Although TF Irf5 expression was lower in the LPD-fed mice, it plays a minimal role in neutrophil functional regulation (Figure S4D).^13^ As such, the role of the tissue microenvironment for neutrophil responses can be neglected in this study. Overall, these findings show that neutrophils mobilizing into the lungs are morphologically and phenotypically “immature,” functionally impaired, and unable to maintain a controlled inflammatory response.

### LPD alters normal neutrophil differentiation and maturation patterns in the BM

Alterations in neutrophil differentiation have been associated with the production and mobilization of aberrant neutrophil populations with altered functionality.^9^^,^^12^^,^^16^^,^^33^ Therefore, we next assessed neutrophil development in the BM to identify intrinsic factors for the higher prevalence of immature neutrophil subsets in the lungs.

**Figure 4:**
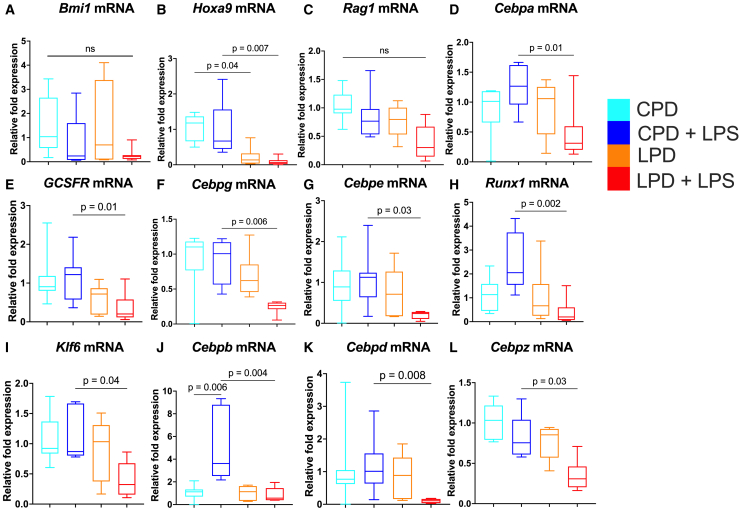
LPD reduces expression of neutrophil-specific genes in BM cells (A–L) qPCR analysis of gene (Bmi1, Hoxa9, Rag1, Cebpa, G-CSFR, Cebpg, Cebpe, RUNX1, KLF6, Cebpb, Cebpd, and Cebpz) expression in CPD, CPD+LPS, LPD, and LPD+LPS. Box and whisker plots of the expression of genes (normalized to expression of Rpl13a in control mice without LPS) encoding hematopoietic, lymphoid, and myeloid development-related genes in total BM cells. Each sample was analyzed in triplicate wells (*n* = 8/group). Significance is determined by non-parametric Kruskal-Wallis test with multiple comparisons test.

**Figure 5:**
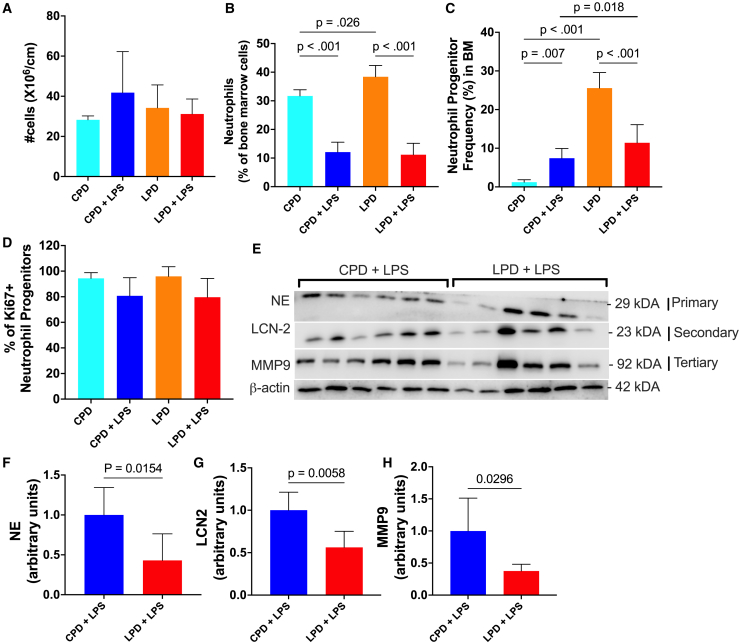
LPD promotes the accumulation of phenotypically and functionally altered neutrophils (A–C) Flow cytometry to compare (A) BM cellularity, (B) the proportion of neutrophils (Ly6G^+^ cells), and (C) the proportion of neutrophil-committed progenitors (ckit^−^CXCR4^−^CD11b^+^Ly6G^+^CXCR2^−^ cells) in the BM. (D) Proportion of Ki67^+^ neutrophil-committed progenitors. Results are expressed as means ± SD (*n* = 6/group) as determined by (A–D) one-way ANOVA. (E) Representative western blot probed for NE, LCN-2, and MMP9 and β-actin in BM neutrophils of LPS-challenged CPD- or LPS-fed mice is shown. (F–H) Quantification of (F) NE, (G) LCN-2, and (H) MMP9 normalized to the amount of β-actin was calculated (*n* = 6/group). Results are expressed as means ± SD as determined by unpaired two-tailed t test analysis.

We first analyzed the gene expression of TFs involved at different stages of granulopoiesis since these TFs are closely linked to proper stepwise differentiation and gain of machinery for neutrophil effector functions. Expression of Bmi1, a self-renewal gene expressed in HSCs, remained unchanged, indicating that maintenance of HSCs is intact in LPD-fed mice with and without LPS challenge, results also seen with flow cytometry (Figure 4A; Figure S5). The expression of Hoxa9, a TF crucial for self-renewal, cell cycle entry, and myeloid differentiation, was consistently lower in LPD-diet fed mice with and without LPS challenge (Figure 4B). Downregulation of Hoxa9 is associated with defects in HSCs and dysregulated downstream myeloid lineage differentiation since Hoxa9 binds with other myeloid TFs to regulate their transcription,^34^ while no impact on B and T cell development is commonly observed. This is consistent with unaltered Rag1 expression, master regulator for lymphocyte commitment, in the LPD host with LPS challenge (Figure 4C). On the contrary, Cebpa, a crucial TF for the initiation of the myeloid lineage program and primary granule gene expression, and its downstream target G-CSFR were lower in LPD-fed mice with LPS challenge (Figures 4D and 4E). Here, although the percentage of CD115^+^ monocytes was reduced in the LPD host, expression of Irf8, the main TF for monocyte differentiation, was unaffected (Figure S6), which led to the sole focus on granulocyte differentiation. Cebpg, a pro-proliferative factor required by early progenitors, was also lower in LPD-fed mice (Figure 4F). Cebpe, which drives the chain of differentiation in post-mitotic neutrophil precursors and contributes to the expression of secondary granules, also showed a similar reduction (Figure 4G). TFs for terminal neutrophil differentiation and production of mature neutrophils and tertiary granules and secretory vesicles, Runx1, Klf6, Cebpb, Cebpd, and Cebpz, were lower in LPS-challenged LPD-fed mice compared to LPS-challenged CPD-fed mice (Figures 4H–4L). Flow cytometry showed that the total cell number and proportion of neutrophils (Ly6G^+^ cells) in the BM remain the same between LPS-challenged LPD- and CPD-fed mice (Figures 5A and 5B; Table S4). Instead, the proportion of early neutrophil-committed progenitors, defined as ckit^−^CXCR4^−^CD11b^+^Ly6G^+^CXCR2^−^ cells, was higher among the BM neutrophils (Figure S7; Figure 5C; Table S4). This was irrespective of expression of Ki67, a marker for proliferation, in these cells (Figure 5D). Therefore, we hypothesized that alterations in the TFs in host fed an LPD post-LPS defined the quality, rather than the quantity, of the neutrophils produced. To assess the extent to which these BM neutrophils were compromised in their maturation due to a consistent reduction in TFs for all stages, we assessed the expression of granule signatures and morphological characteristics in these cells. Ly6G^+^ BM cells from LPS-challenged LPD-fed mice consistently had lower NE, LCN-2, and MMP9 protein expression, data consistent with altered neutrophil differentiation and accumulation of phenotypically and functionally immature neutrophils (Figures 5E–5H). In line with the qPCR data, LPD feeding alone did not impact the expression of these granule proteins (Figures S8A–S8E). Transmission electron microscopy also revealed more nuclear lobulation and neutrophils with smaller cell size in the LPD-fed mice, pointing to an accumulation of these atypical “hypersegmented” immature neutrophils in the BM (Figures S8F and SFG). Here, expression of Lamin-B2, predominantly expressed in mature neutrophils, remained unaltered, highlighting that these changes define immature, rather than mature, neutrophils (Figures S8H and S8I). Altogether, these data indicate global aberrations in neutrophil functions related to differentiation changes and the accumulation of “immature” neutrophils in the BM and lungs of young mice exposed to an LPD.

### Protein restriction impairs mitochondrial ATP production and dynamics in BM neutrophils

**Figure 6:**
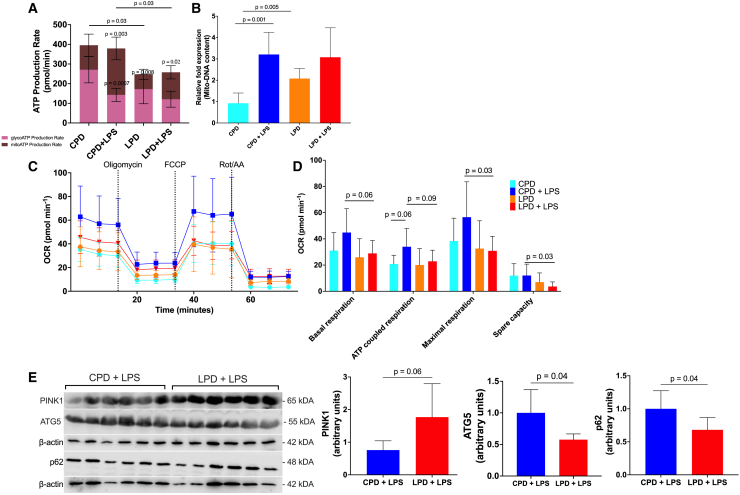
LPD impacts BM neutrophil metabolic requirements (A) ATP production from both glycolysis and mitochondrial respiration was measured in BM neutrophils using the Seahorse ATP Rate Assay (*n* = 6/group). Results are expressed as means ± SD as determined by two-way ANOVA with Tukey’s multiple comparisons test. (B) Mitochondrial DNA (mtDNA) content was measured by qPCR and normalized to expression of β-globin in control mice without LPS in BM neutrophils. Each sample was analyzed in triplicate wells; each symbol represents an individual mouse. Results are expressed as means ± SD (ΔΔCt); *n* = 6/group. Significance is determined by one-way ANOVA. (C) Mitochondrial stress test in BM neutrophils (1.5 μM oligomycin, 2.5 μM FCCP, 1 μM Rotenone, and 1 μM antimycin A) (*n* = 6/group). (D) Oxygen consumption rate (OCR) was measured under basal conditions and in response to indicated drugs in BM neutrophils (*n* = 6/group). (C and D) Results are expressed as means ± SD from multiple independent experiments as determined by two-way ANOVA with Tukey’s multiple comparisons test or unpaired two-tailed t test analysis. (E) Representative western blot probed for PINK1, ATG5, p62, and β-actin in BM neutrophils of CPD- or LPD-fed LPS-challenged mice is shown (left). Quantification of PINK1 (right), ATG5 (middle), and p62 (left) normalized to the amount of β-actin was calculated (*n* = 6/group). Results are expressed as means ± SD as determined by unpaired two-tailed t test analysis.

Cellular metabolism is known to modulate cellular differentiation. In this regard, neutrophil differentiation is largely dependent on energy metabolism through mitochondrial respiration, while glycolysis and the pentose phosphate pathway regulate downstream effector functions.^16^ Therefore, we next focused on the metabolic processes known to be essential for neutrophil differentiation. We hypothesized that metabolic perturbations in mitochondrial respiration could be associated with the accumulation of aberrant immature neutrophil subsets in the BM. We first measured ATP production in BM neutrophils and observed that neutrophils collected from mice from the CPD and LPD groups but not exposed to LPS were highly glycolytic, in keeping with the literature on mature neutrophils that are dominant in the non-LPS conditions (Figure 6A). Glycolytic ATP levels were lower in LPD- compared to CPD-fed mice (Figure 6A), where immature neutrophils were higher (Figure 5C). LPS challenge stimulated a metabolic shift toward mitochondrial ATP production in CPD- and LPD-fed BM neutrophils (Figure 6A). However, we found total and specifically mitochondrial ATP production to be lower in the neutrophils of LPS-challenged LPD-fed mice compared to CPD-fed mice (Figure 6A). To further understand the mechanism of lowered mitochondrial ATP in LPD-fed mice, we examined mitochondrial quantity and quality. Through mitochondrial DNA (mtDNA) quantification, we confirmed that the mitochondrial content was unaffected between LPS-challenged CPD and LPD neutrophils, although higher mtDNA levels were present with LPD feeding alone (Figure 6B). This suggested impaired mitochondrial fitness in BM neutrophils in LPD-fed mice possibly related to perturbed differentiation and accumulation of immature neutrophils (Figure 5C). We further utilized the Seahorse extracellular flux analysis to measure the oxygen consumption rate (OCR) as a key indicator of mitochondrial respiration and activity in real time in the presence of inhibitors for the electron transport chain such as oligomycin, FCCP, and rotenone/antimycin A. BM neutrophils from LPS-challenged LPD-fed mice possessed significantly lower mitochondrial basal respiration, ATP turnover, and mitochondrial maximal and spare OCR capacities than BM neutrophils from LPS-challenged CPD-fed mice, indicative of reduced mitochondrial function (Figures 6C and 6D). Mitophagy is a central process guarding mitochondrial quality through the removal of damaged mitochondria and maintaining mitochondrial fitness for oxidative metabolism. We quantified proteins of the Parkin-PINK1 signaling cascade, which is responsible for the degradation of damaged mitochondria by polyubiquitination of proteins present on the surface of damaged mitochondria to induce autophagosome recruitment.^35^ Here, higher expression of PINK1, and lower ATG5 and p62, both commonly used in immunometabolism studies,^36^^,^^37^ indicated higher mitochondrial damage and lower autophagy (Figure 6E), further pointing toward a potential pathway for compromised mitochondrial quality control and metabolic output. Here, mTOR activity was also lower, as measured by the phosphorylated p70S6K-to-total p70S6K ratio, in LPS-challenged neutrophils from the LPD host (Figure S9). Altogether, our findings suggest that the accumulation of phenotypically and functionally immature neutrophils in the BM and lungs is related to impairments in neutrophil mitochondrial function.

### NAM treatment improves LPD-driven metabolic perturbations for neutrophil development in the BM and downstream effector functions

**Figure 7:**
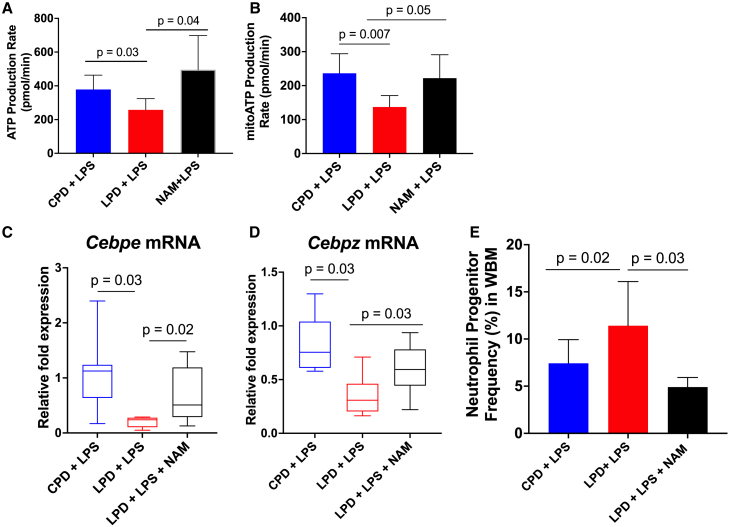
NAM supplementation restores ATP production and transcriptomic profiles in LPD BM neutrophils (A and B) ATP production (A) and, more specifically, mitochondrial respiration (B) were measured in BM neutrophils using the Seahorse ATP Rate Assay (*n* = 6/group). Results are expressed as means ± SD as determined by two-way ANOVA with Tukey’s multiple comparisons test or unpaired two-tailed t test analysis. (C and D) Box and whisker plots of qPCR analysis of (C) Cebpe and (D) Cebpz normalized to expression of Rpl13a in total BM cells. Significance is determined by non-parametric Kruskal-Wallis test with multiple comparisons test (*n* = 8/group). (E) Flow cytometry to compare the proportion of neutrophil-committed progenitors (ckit^−^CXCR4^−^CD11b^+^Ly6G^+^CXCR2^−^ cells) in the whole BM (WBM) of NAM-treated mice. Results are expressed as means ± SD (*n* ≥ 3/group) as determined by one-way ANOVA with Tukey’s multiple comparisons test.

We have previously shown in humans and pre-clinical models of severe malnutrition that tissue-specific reduced NAM adenine dinucleotide (NAD^+^) bioavailability is associated with mitochondrial dysfunction and altered tissue homeostasis.^19^^,^^22^^,^^23^ Modulation of NAD^+^ synthesis through its precursor, NAM, restored these tissue-specific alterations. Therefore, we next assessed the metabolic fitness of BM neutrophils with NAM supplementation and found a complete rescue in mitochondrial-driven ATP function compared to LPS-challenged LPD neutrophils (Figures 7A and 7B). In addition, NAM was sufficient to partially or completely upregulate the transcriptomic profiles of neutrophil-development genes (Figures 7C and 7D) that were found to be lower in the LPS-challenged LPD diet group and correct the LPD-mediated expansion of immature neutrophils in the BM (Figure 7E).

## Discussion

Malnutrition is associated with an increased susceptibility to infection related to inadequate immune responses and exaggerated inflammatory processes that contribute to elevated morbidity and mortality.^1^^,^^2^^,^^3^^,^^38^ Here, we studied the role of neutrophil differentiation, function, and association with neutrophil metabolism in a mouse model of malnutrition. We uncovered the accumulation of immature neutrophils with impaired functional capacity in LPS-challenged LPD-fed mice, which was associated with signs of higher mitochondrial damage and diminished mitochondrial ATP levels. This may, in turn, disturb neutrophil terminal differentiation through an altered transcriptional landscape, leading to an accumulation of phenotypically and functionally atypical immature neutrophils in the BM and peripheral tissues. Although the enhancement of these atypical immature neutrophils in both the BM and lungs indicates a compensatory mechanism, there is higher bacterial burden and lung immunopathology, indicating impaired neutrophil responses, and worse prognosis in the protein-malnourished host. This is characteristic of phenotypes we have commonly observed in children with severe malnutrition where hyperinflammation,^2^^,^^3^^,^^39^^,^^40^ mitochondrial derangements,^3^^,^^19^ and neutrophil-mediated damage may intersect to contribute to infectious disease severity and mortality in these children. This connection, to our knowledge, has not been explored in murine models of malnutrition and associated inflammatory disease prior to this study. In our study, in LPD-fed mice, supplementing with NAM provides a therapeutic benefit to ameliorate perturbations in neutrophil mitochondrial ATP production and neutrophil differentiation.

Our study demonstrated impaired neutrophil responses, especially NADPH-oxidase ROS production, bacterial killing capacity, elevated pulmonary pro-inflammatory cytokine expression, and prolonged inflammation, in our malnutrition mouse model. These findings are consistent with the limited data on impaired neutrophil responses in childhood malnutrition,^5^^,^^6^^,^^7^^,^^38^ although the mechanism for this was not elucidated in these prior studies. A recently published study similarly showed that calorie restriction, not leading to malnutrition, in mice challenged with virulent tuberculosis bacteria had downregulated expression of genes for neutrophil activity and reduced lung damage, while animals fed *ad libitum* had elevated infection and associated pathology.^18^ Unlike the previous studies in childhood malnutrition conducted solely in peripheral blood, which does not truly capture tissue-specific neutrophil functions, we explicitly show that NADPH-oxidase-dependent ROS production is altered in LPD neutrophils and explore a specific mechanism for this dysfunction.

We identified, for the first time, atypical immature neutrophils that constitute the neutrophil population in the inflamed lungs of LPD-fed mice. We show that the presence of these immature neutrophils in the lungs is associated with local, i.e., lung damage, pulmonary vascular permeability, and edema, and systemic damage that was observed in LPS-challenged LPD-fed mice. Several recent publications have emphasized that the mobilization of distinct types of neutrophils with altered functions has a role in inflammatory disease outcomes. Similar to our findings, in a pre-clinical model of bacterial infection, the accumulation of immature neutrophils was associated with reduced expression of NADPH oxidase subunits and, therefore, ROS production and higher bacterial survival in lungs, despite elevated phagocytosis.^41^ In patients with sepsis, the higher prevalence of immature neutrophils with immunosuppressive functions in the blood is associated with a higher sepsis severity scores and poor prognosis.^42^ Similarly, in another study, IL-1R2^+^ immature neutrophils from patients with sepsis were associated with more severe disease and a higher risk of early mortality.^43^ Indeed, an enhanced and incomplete granulopoietic response in the BM in multiple pre-clinical models of inflammatory disease states was found to lead to an accumulation of these suboptimal neutrophils with an altered capacity for phagocytosis and bacterial killing in blood and peripheral tissues.^13^^,^^16^^,^^33^^,^^41^

In our study, we found the population of immature neutrophils, ckit^−^CXCR4^−^CD11b^+^Ly6G^+^CXCR2^−^, to originate from the BM, where its presence was also higher and reprogrammed toward phenotypic changes that induce inflammation. These alterations were thought to occur because of reduced transcriptional regulators and subsequent expression of granule proteins, not simply due to the depletion of BM mature neutrophils, which were unaltered in our study. In fact, only the composition of the neutrophil subpopulations was altered in the BM both pre- and post-LPS in LPD-fed mice and not the total neutrophil numbers. A recently published study similarly found impaired myelopoiesis in the blood of children with SAM, with reduced levels of MPO in stool.^38^ Here, we show through qPCR that all the major TFs for neutrophil development were reduced in the LPD-fed mice with LPS challenge in the BM, a finding that has not been reported before even in animals fed normal chow. Similarly, the expression of primary, secondary, and tertiary granule proteins regulated by these TFs was also reduced, in line with reduced expression and activity of the TFs. As the granule composition is strongly dependent on stepwise neutrophil differentiation and affects effector functions, dysregulated neutrophil differentiation in the BM could explain the association between the accumulation of immature neutrophils and impaired responses in the LPD host. In addition, a recent study defined CD177^lo^CD101^lo^CD62L^hi^ immature hypersegmented atypical neutrophils in the blood, endowed with higher oxidative stress, phagocytosis, and procoagulant features for aggravated stroke pathology in aging.^33^ This is to say that although our study has only focused on the characterization of immature neutrophils based on well-defined surface markers, it is possible that an increase in atypical immature neutrophils contributes to the expansion in BM and lungs and noxious outcomes, which remains to be explored further.

Although previous studies have highlighted the role of molecular regulators in emergency granulopoiesis,^44^^,^^45^^,^^46^ the metabolic pathway involved in this process was not shown prior to this study. This study is the first, to our knowledge, to explore immunometabolism in BM neutrophils from LPD-fed and LPS-challenged mice, while previous studies have only focused on the metabolism of neutrophils in healthy mice and humans or in genetically modified mice. Our findings suggest that the mitochondria could instruct neutrophil development in the BM and dictate their peripheral responses upon LPS challenge as well, as has been reported recently.^47^

In fact, our study shows, through extracellular flux analyses, metabolic reprogramming from glycolytic to mitochondrial-dependent ATP production in LPS-challenged BM neutrophils. This metabolic shift may be driven by higher BM fat lipolysis and higher availability of substrates for FAO with inflammatory challenge, as has already been reported.^48^^,^^49^ In turn, FAO may modulate mitochondrial ATP generation and rapid cellular differentiation to meet the higher neutrophil demand to protect the host from inflammatory challenge.^16^^,^^48^ Given this, impaired mitochondrial respiration and ATP depletion, due to depletion of free fatty acids, lead to the accumulation of phenotypically immature populations of neutrophils that have a reduced capacity for antimicrobial functions.^16^ Exogenous treatment with a mixture of saturated and unsaturated free fatty acids here restores OXPHOS and neutrophil differentiation.^16^ In our study, LPS-challenged LPD BM neutrophils showed reduced mitochondrial ATP production compared to CPD neutrophils. We previously reported the depletion of long-chain lipids (lysophospholipid, sphingolipid, and phospholipid species) for FAO in malnourished mice and children.^3^^,^^22^ Although a detailed examination of the altered fuel sources is required in LPD neutrophils, limited substrate availability in LPD BM neutrophils for mitochondrial respiration may underlie the perturbed differentiation and accumulation of neutrophil progenitors in the BM that are unable to carry out optimal antimicrobial responses. This remains to be studied in greater detail.

Children with severe wasting have lower levels of lean mass and peripheral fat mass (adipose tissue).^50^ Importantly, low discharge lean and peripheral fat mass has been reported to be independently associated with death and hospital readmission in children treated for complicated severe acute malnutrition in Zimbabwe and Zambia.^50^ Reduced fat mass affects levels of adipokines, a group of cell signaling molecules. Children with severe malnutrition also have lower levels of the adipokine leptin,^2^^,^^39^ which have been reported to be associated with mortality in this context.^39^ Most immune cells express the leptin receptor to directly alter immune responses, and the reduction in leptin levels may play a role in changes in neutrophil biology. Leptin can regulate the inflammatory response through tumor necrosis factor alpha (TNF-α)-dependent neutrophil activation and regulation of its capacity for ROS and cytokine production.^51^^,^^52^ Additionally, TNF-α/TNFR1- and CXCL1-dependent signaling pathways are important for leptin-induced neutrophil migration *in vivo.*^53^ Leptin is also capable of blunting the exacerbated pro-inflammatory cytokine (IL-1β , IL-6, TNF-α) and tissue damage response during endotoxemia, where leptin-deficient mice are more susceptible to LPS-induced death.^54^^,^^55^^,^^56^ Other adipokines, including adiponectin, are also known to play a role in neutrophil-mediated immunity.^57^^,^^58^ Therefore, leptin and other adipokines provide an excellent direction for future work to further enhance our understanding of the impact of malnutrition on neutrophil development and function. Mitochondrial metabolites also function as signaling molecules to control chromatin modifications of proteins and, thus, cellular function and fate.^59^^,^^60^ Therefore, the link between mitochondrial metabolic substrates and epigenome remodeling and transcriptional regulators significantly impacted in our LPD mouse model of malnutrition should also be further explored.

In addition to limited substrate availability, aberrant mitochondrial dynamics alter uptake and utilization of available metabolic substrates and disrupt energy metabolism.^61^ Mitochondrial quality control maintains mitochondrial homeostasis and function. Indeed, upregulation of mitophagy machinery, such as through the PINK1-PARKN pathway Sirt3, by the mTOR signaling pathway is implicated in reducing mitochondrial stress and improving mitochondrial quality through clearance of damaged mitochondria in rodent studies.^36^^,^^62^^,^^63^ Alterations in the activity of the autophagy pathway leading to an accumulation of dysfunctional mitochondria are implicated in a wide range of diseases.^35^^,^^64^ In fact, our group recently reported that livers and gut of mice fed an LPD had decreased expression of proteins for autophagy, LC3B-II, and accumulation of damaged and dysfunctional mitochondria that were associated with impaired hepatic and gut function.^22^^,^^23^ Here, treatment with NAM, a form of vitamin B3, improved mitochondrial quality and hepatic and gut function through mitophagy activation.^22^^,^^23^ The effect of malnutrition on autophagic machinery and mitochondrial quality control in LPS-challenged BM neutrophils, where we found higher PINK1 and lower ATG5 and p62, remains to be explored further and may independently drive neutrophil activation and pathology.

In this study, in an LPD mouse model, we show for the first time that NAM was sufficient to restore mitochondrial function and improve the neutrophil differentiation program that was associated with impaired neutrophil antimicrobial responses. An earlier study showed that NAM mediated NAD^+^-dependent sirtuin-1 activation, and subsequent binding and activation of Cebpa and Cebpb ultimately regulate neutrophilic differentiation.^65^ Additionally, NAM has been shown to alter the transcriptional profiles of myeloid precursor cells.^36^^,^^63^ The exact mechanism that connects these processes in our model remains to be further explored.

In conclusion, this study has demonstrated compelling evidence for a role of mitochondrial function in neutrophil biology in mice fed an LPD. Particularly, diminished mitochondrial quality control processes and ATP production in BM neutrophils may arrest sequential differentiation, resulting in the mobilization of immature neutrophils to exacerbate lung inflammation in LPD-fed mice. A better understanding of the mechanisms by which the mitochondria confer protection for neutrophil homeostasis in systemic inflammation may facilitate the discovery of additional therapeutic targets for clinical use in the context of malnutrition.

### Limitations of the study

Our study has several limitations. Although we identified higher lung damage through an elevated W/D ratio, the dynamics of edema formation remains to be explored in greater detail. The interplay between IL-1β and edema formation will be critical to explore here, especially since IL-1β secretion is known to influence endothelial cell permeability.^66^ Although not explored in this study, the LPS-driven acute inflammatory response is self-limited and resolved within a few hours. It is possible that the time course of the acute inflammatory response is extended from initiation to the resolution phase in the malnourished host and requires investigation. We also did not investigate the hyperinflammatory response through assessment of serum cytokine profiles in this study more closely due to difficulty in obtaining sufficient serum samples from the LPD-fed mice. Additionally, we did not explore the role of other innate immune cell types in the acute response to LPS in great detail, nor the function of neutrophils at the primary site of LPS injection, in both earlier time points as well as in distant tissues. IRF5, which was reduced in the current study, may also be relevant for macrophage functions such as phagocytosis and cytokine generation across tissues and should be explored further. As malnutrition directly leads to changes in adipose tissue and levels of adipokines, and given the known roles of different adipokines on neutrophil biology, it would have been interesting to explore this further. Therefore, as we did not study the role of adipokines in the effect of an LPD on neutrophil development and function, this represents a limitation of our study. In this study, we only focused on a 2-week protein restriction in male mice at 5 weeks of age to study the impact of early exposure on early life outcomes. Therefore, the role of long-term protein deficiency as well as of other dietary patterns, such as multiple micronutrient deficiencies, that closely mimic real-life circumstances of children living with malnutrition for a prolonged period of time remained unexplored. Future work would also benefit from examining sex differences in this LPD model of malnutrition. It also remains unclear as to how reduced mitochondrial function directly impacts neutrophil differentiation, which is the focus of our future work.

## STAR★Methods

### Key resources table

**Table:** 

REAGENT or RESOURCE	SOURCE	IDENTIFIER
**Antibodies**		

Anti-mouse CD117 (ckit) (Clone 2B8) - PerCP-Cy5.5	BD Biosciences	Cat# 560557; RRID: AB_1645258
Anti-mouse Ly6G (Clone 1A8) - FITC	BD Biosciences	Cat# 551460; RRID: AB_394207
Anti-mouse CD182 (CXCR2) (Clone SA044G4) - Alexa Fluor 647	Biolegend	Cat# 149306; RRID: AB_2565694
Anti-mouse CD184 (CXCR4) (Clone 2B11/CXCR4) - PE	BD Biosciences	Cat# 551966; RRID: AB_394305
Anti-mouse CD115 (CSF-1R) (Clone AFS598) - Brilliant Violet 605	BD Biosciences	Cat# 750892; RRID: AB_2874988
Mouse anti-Ki67 (Clone B56) - PE-Cy7	BD Biosciences	Cat# 561283; RRID: AB_10716060
Anti-CD11b (Clone M1/70) - Brilliant Violet 421	BD Biosciences	Cat# 562605; RRID: AB_11152949
Anti-mouse CD16/32 (FcgRIII/II) (Clone 2.4G2) - Purified (Mouse BD Fc Block)	BD Biosciences	Cat# 553142; RRID: AB_394657
Fixable Viability Stain 620	BD Biosciences	Cat# 564996; RRID: AB_2869636
Mouse Anti-MPO Antibody (Clone 2C7)	Abcam	Cat# ab25989; RRID: AB_448948
Rabbit Anti-Neutrophil Elastase antibody	Abcam	Cat# ab68672; RRID: AB_1658868
Rabbit Anti-Lipocalin-2/NGAL antibody [EPR21092]	Abcam	Cat #ab216462
Rabbit Anti- MMP-9 Antibody (Clone C-20)	Abcam	Cat# ab38898; RRID: AB_776512
beta Actin Monoclonal Antibody	Invitrogen	Cat #AM4302
Rabbit Anti-P62	Novus Biologicals	NBP1-48320B
Rabbit Anti-PINK1	Novus Biologicals	BC100–494
Rabbit Anti-Lamin B2	Abcam	ab151735; RRID: AB_2827514
Rabbit p70S6K	Cell Signaling	2708; RRID: AB_390722
Rabbit p-p70S6K	Cell Signaling	9205; RRID: AB_330944
Rabbit ATG5	Cell Signaling	2630; RRID: AB_2062340
Goat Anti-Mouse	Invitrogen	62–6520
mouse anti-rabbit IgG-HRP	Santa Cruz Biotechnology	Cat# sc-2357; RRID: AB_628497

**Bacterial and virus strains**		

LPS (derived from *Escherichia coli* O55:B5)	Sigma-Aldrich	Cat# L2880
Salmonella enterica ssp. enterica serovar Typhimurium (SL1344)	Brumell lab^68^	N/A

**Chemicals, peptides, and recombinant proteins**		

Dihydrorhodamine 123	ThermoFisher Scientific	Cat# D23806
DAPI	Abcam	Cat# 228549
Transcription Factor Buffer Set	BD Bioscience	Cat# 562725
Brilliant Stain Buffer Plus	BD Bioscience	Cat# 566385
Phorbol 12-myristate 13-acetate (PMA)	Sigma-Aldrich	Cat# P8139
Trypan Blue	Sigma-Aldrich	Cat# T8154
TRIzol Reagent	ThermoFisher Scientific	Cat# 15596018
Collagenase A	Sigma-Aldrich	Cat# 10103578001
Bovine Serum Albumin Powder	Tocris	Cat# 5217
Bovine Serum Albumin solution	Sigma-Aldrich	Cat# A9576
Sodium Chloride	Sigma-Aldrich	Cat# S9888
RPMI	Gibco	Cat# 11875093
Advanced qPCR Mastermix Super Green	Wisent	Cat# 800-431-UL
30% Acrylamide/Bis Solution	Bio-Rad	Cat# 1610158
CountBright Absolute Counting Beads, for flow cytometry	ThermoFisher Scientific	Cat# C36950
Tween 20	Sigma-Aldrich	Cat# P1379
Seahorse XF RPMI	Agilent	Cat# 103576-100

**Critical commercial assays**		

Direct-zol RNA MiniPrep Kit	Zymo Research	Cat# R2052
Neutrophil Isolation Kit, mouse	Miltenyi Biotec	Cat# 130-097-658
qScript cDNA Synthesis Kit	QuantaBio	Cat# 101414-098
LS Columns	Miltenyi Biotec	Cat# 130-042-401
ECL™ Prime Western Blotting System	Sigma-Aldrich	Cat# RPN2232
Pierce™ BCA Protein Assay Kit	Thermo-Fisher Scientific	Cat# 23227
Seahorse XF Cell Mito Stress Test Kit	Agilent	Cat#103015
Seahorse XF Real-Time ATP Rate Assay Kit	Agilent	Cat# 103592

**Deposited data**		

Raw data for 7 main figures and 8 supplementary figures, and Western Blot Full Gels	Mendeley Data	https://data.mendeley.com/preview/r8ytz983hg?a=5edbef76-f1d4-411e-bb00-86fc8241c8cc

**Experimental models: Organisms/strains**		

Mouse: C57BL/6	Jackson Laboratories, Bar Harbor, Maine, USA	N/A

**Oligonucleotides**		

Table S3	Integrated DNA Technologies	N/A

**Software and algorithms**		

FACSDiva software	BD Biosciences	N/A
FlowJo 10 Software	TreeStar	http://flowjo.com/
GraphPad Prism 9	GraphPad Software	http://www.graphpad.com
Image Studio Lite	LI-COR Biosciences	https://www.licor.com/bio/image-studio-lite/
ImageJ	National Institutes of Health	https://imagej.nih.gov
Volocity	Quorum Technologies Inc	https://www.volocity4d.com
Seahorse Analytics	Agilent	https://www.agilent.com

**Other**		

CPD	Envigo	RX: 2576206; TD: 180483
LPD	Envigo	RX: 2576185; TD: 180481

### Resource availability

#### Lead contact

Further information and requests for resources and reagents should be directed to and will be fulfilled by the lead contact, Amber Farooqui (amberfarooqui@hotmail.com).

#### Materials availability

This study did not generate new unique reagents.

#### Data and code availability

• This paper does not report original code.
• Any additional information required to reanalyze the data reported in this paper is available on Mendeley. The DOI is listed in the key resources table.

### Experimental model and study participant details

#### Mice and ethics statement

All mouse experiments were approved by and performed in accordance with the Animal Care and Use Committee guidelines (protocol number: 1000058060) at Lab Animal Services (LAS) Facility of SickKids, Toronto. A breeding colony of specific pathogen free C57Bl/6J mice was obtained from The Jackson Laboratory (Bar Harbor, Maine USA). At three weeks post-partum, male mice were weaned from their dams. Weight-matched weanling C57Bl/6J male mice (21-days old) were randomized into one of two diet groups for a period of two weeks: (1) a control (RX: 2576206; TD: 180483), and (2) a low-protein (RX: 2576185; TD: 180481) diet made by Envigo Teklad Diets (Madison, WI). The diet composition is provided in Table S1. In a subset of animals, single i.p injection of 400 mg/kg of NAM was also administered. All animals were group housed in specific pathogen-free (SPF) conditions and temperature-controlled environment (22 ±2°C), under a 12-h light-dark cycle with food and water *ad libitum* throughout the study period in the Lab Animal Services (LAS) Facility of SickKids, Toronto. Body weight, and food/water was recorded for subsequent days and clinical scoring (Table S2) was determined to assess disease severity during the experimental period to minimize suffering to the animals. Animals that exceeded the humane endpoint for weight loss during the experimental period prior to the day of sacrifice were excluded from the study.

### Method details

#### LPS-induced systemic inflammation

Systemic inflammation was induced by a single intraperitoneal (i.p) injection of LPS (*Escherichia coli* 055: B5; Sigma-Aldrich; 4 mg/kg) diluted in 100 μl PBS in mice from both diet groups. Mice were then humanely sacrificed 24 h post-challenge with isoflurane, tissues harvested, and cells collected as detailed below.

#### *In vivo* Salmonella infection and quantification of bacterial loads

For infection of mice, wildtype *Salmonella enterica* serovar *Typhimurium* strain SL1344, Brumell lab^67^^,^^68^ was used. Strain S2337, which was originally isolated from a calf with salmonellosis, is the parental strain of SL1344 and is highly virulent in cattle, pigs, chickens, and mice.^69^ SL1344 was grown to log phase in Luria-Bertani (LB) broth at 37°C for 4–5 h without antibiotics to an optical density (OD) at 600 nm of 1.03 (2X10^8^ colony-forming unit (CFU)/mL). Mice were infected through oral gavage with 50 μ l of inoculum containing a total of 10^7^ CFU bacteria in PBS and were sacrificed 48 h later. For bacterial load determination, liver, lung, and spleen were harvested, weighed, and homogenized in 1 mL PBS, plated in appropriate dilutions on LB agar plates, and incubated at 37°C overnight. The number of colonies were counted the next day and plotted as a CFU count.

#### Single-cell suspension and neutrophil isolation

All samples in this study were taken from mice sacrificed at the same time, due to the known intrinsic changes that influence neutrophil numbers and phenotypes throughout the day.^70^^,^^71^ Peripheral blood was collected by cardiac puncture using a 25-gauge needle, heparinized syringe in a 1.5 mL Eppendorf tubes containing heparin and then fixed in 4% paraformaldehyde in PBS for 30 min at 4°C. Blood cells were later subjected to red blood cell (RBC) lysis with a hypotonic (0.2% NaCl) followed by hypertonic (1.6% NaCl) lysis solution at room temperature (RT) in Milli-Q water. For bone marrow (BM) cells isolation, muscle tissues were removed from the bones and mouse femur and tibia were flushed using a 25-gauge needle in RPMI (Gibco) containing 2 mM EDTA (Invitrogen) and 10% fetal bovine serum (FBS) and filtered through a 70- μ m cell strainer to obtain single-cell suspensions. To prepare single-cell suspension from lung tissues, whole lung tissue was digested in 1.5 mg/mL Collagenase A (Roche) for 30 min at 37°C. RPMI containing 10% FBS and 2mM EDTA was later added to inhibit the digestion process and homogenized into single-cell suspensions using 70 μm cell strainer and syringe plungers. Finally, peritoneal cavity exudate cells were harvested by three successive washes with 3 mL RPMI +2 mM EDTA +10% FBS. Cells were centrifuged at 4°C for 10 min at 300*g* and resuspended in 1 mL RPMI containing 10% FBS and 1% penicillin/streptomycin. Cells were counted and viability was checked with Trypan blue using a hemocytometer and only samples with viability >90% were used for subsequent experiments. Neutrophils were enriched, where indicated, by negative selection using the Neutrophil Isolation Kit (Miltenyi Biotec) with magnetic-activated cell sorting (MACS) buffer (0.5% BSA and 250 mM EDTA in PBS) prepared in-house.

#### Cell staining for flow cytometry

All fluorochrome - conjugated anti-mouse monoclonal antibodies for flow cytometry were purchased from BD, or Biolegend, and titrated prior to use. Mouse cells were stained in FACS staining buffer (PBS +3% FBS; produced in house) on ice at a density of 10–20 million cells. Prior to surface staining, cells were blocked with rat anti-mouse CD16/32 (2.4G2; BD Biosciences) antibody on ice for 5 min to stain and block the FcγII and FcγIII receptors to prevent nonspecific binding. To assess the mouse neutrophils, PerCP/cy5.5-conjugated anti-CD117, FITC-conjugated anti-Ly6G, and BV421- conjugated anti-CD11b antibodies were added to the cells and incubated for 30 min at 4°C in the dark. Cells were washed and resuspended in FACS staining buffer before acquisition. Absolute cell counts were calculated using fluorescent count beads (CountBright Absolute Counting Beads, ThermoFisher Scientific; cat# C36950) according to the manufacturer’s protocol. Cell phenotyping was performed on LSRII-CFI VBYR (BD Biosciences) equipped with violet, blue, yellow/green, and red lasers using FACSDiva software and data was subsequently analyzed with the FlowJo (Tree Star) and FCS Express 7 software. Fixable Viability Stain 620 (BD Horizon) was used to discriminate between dead and viable cells and SSC-A and SSC-H discrimination was used to exclude doublet cells. Gating for each marker was determined using single stained controls, that included only one antibody at a time, for each color used in the experiment.

#### RNA extraction, cDNA synthesis and quantitative PCR (qPCR)

Total RNA was extracted from total BM cells and whole lung tissue (right and left) using TRIzol Reagent (Thermo Fisher) and Direct-zol RNA MiniPrep Kit (Zymo Research), which included DNAase digestion, as per manufacturer’s instructions. The RNA concentration was quantified using a Nanodrop spectrophotometer (Thermo Scientific). 1 μ g of RNA was reverse transcribed using qScript cDNA synthesis kit (Quantabio) as per manufacturer’s instructions and resulting cDNA was stored at −20° C. qPCR was performed in triplicates in a 384-well PCR plate loaded with cDNA, primer and advanced SYBR green qPCR mastermix (Wisent) using CFX384 Touch Real-Time PCR Detection System (Bio-Rad) and gene expression data was analyzed using the 2^− (ΔΔCt)^ method. Primer sequences are listed in Table S3. Gene expression was normalized relative to Ribosomal protein L3A (*Rpl13A*) or Beta-actin (*ActinB)* expression and expressed as mRNA expression relative to BM cells of CPD group without LPS. All primer sequences were designed and verified using the Basic Local Alignment Search Tool (BLAST; blast.ncbi.nlm.nih.gov/Blast.cgi). All primer sequences listed below were purchased from Integrated DNA Technologies (IDT; Coralville, Iowa USA).

#### Measurement of mitochondrial DNA

Total genomic DNA was extracted from primary mouse BM neutrophils with a Qiagen DNA Mini kit as per manufacturer’s instructions. The DNA concentration was quantified using a Nanodrop spectrophotometer (Thermo Scientific), qPCR and data analysis was performed as described above.

#### Histology

For histological analyses, mouse lung tissues were fixed in 10% formalin for 1 week at RT. Tissues underwent dehydration, clearing and infiltration steps in an automated processor. Tissues were then paraffin-embedded and lung sections were cut (5-μm-thick sections) for hematoxylin and eosin (H&E) staining, dehydrated and mounted using a standardized protocol. Digital light microscopic images were acquired and assessed for inflammation according to a published protocol by researchers blinded to the experimental groups.^72^ Additionally, the lung wet dry ratio (W/D ratio) was used to evaluate the severity of pulmonary damage and edema. Briefly here, the right lung was dissected, and the wet weight was measured. The lung was then placed in an incubator at 55°C for 72 h to obtain the dry weight.

#### Intracellular ROS assay

Purified neutrophils (2X10^5^ cells/well) were seeded in a black clear bottom 96-well plate and incubated with 10 μ M of cell permeable Dihydrorhodamine 123 (DHR123) probe (ThermoFisher, cat# D23806) in RPMI for 30 min at 37°C. The cells were then stimulated with 50 nM Phorbol 12-Myristate 13-Actetate (PMA) (Sigma-Aldrich). NADPH oxidase inhibitor DPI was added 1 h prior to stimulation with PMA and incubated at 37°C. Relative fluorescence intensity (RFI) was measured by a fluorescence microplate reader (Molecular Devices Fluorescence Plate Reader) at 507/529 nm (excitation/emission) at 30-min intervals up to 4 h to quantify intracellular ROS production, after PMA stimulation.

#### Bacterial killing assay

Purified neutrophils (1X10^5^ cells/well) were infected for 3 h with *E. coli* at MOI of 10/neutrophil at 37°C and then lysed with 1% Triton X-100 buffer. The lysate was serially diluted, plated on LB agar plates, and incubated at 37°C overnight. The number of colonies were counted the next day and plotted as a CFU per mL.

#### If & image analysis

For analyses of cell nuclear morphology, purified neutrophils were plated on poly-*d*-lysine hydrobromide (Sigma) coated chamber slides (Ibidi) and left 30 min to adhere. Cells were then fixed with 4% paraformaldehyde in PBS for 15 min and rinsed with PBS. Samples were permeabilized (PBS with 0.2% saponin and 10% of goat serum) for 30 min at RT. After washing samples with PBS, cells were stained DAPI (1:1000; abcam), mounted in Dako Faramount Aqueous Mounting Medium (Agilent), and stored at RT in the dark overnight. Images were acquired on a ZEISS LSM 980 laser scanning confocal microscope (Zeiss) with a 63× oil immersion objective and Zen 3.6 acquisition software. Images were assessed by researchers blinded to the experimental groups to determine the percentage of myelocyte, metamyelocyte, and band and segmented neutrophils in each group. Myelocytes (MC) are characterized by a round nucleus, metamyelocytes (MM) by kidney-shaped nuclei, band cells (BC) with a band-shaped and segmented neutrophils with a segmented nucleus.

#### Western blotting

Lung tissues or purified bone marrow neutrophils were sonicated on ice in tissue or cell lysis buffer (Thermo Scientific), respectively, supplemented with a cocktail of protease inhibitors (Sigma). Protein concentration was measured using BCA Protein Assay Kit (Thermo Scientific) according to the manufacturer’s protocol. 20 μ g protein per sample was separated on 10%–16% SDS-PAGE and transferred to PVDF membrane (Millipore) by wet western blot, Membranes were blocked 1 h at RT in TBS-Tween 0.1% containing 3% BSA. Membranes were blotted for primary antibodies overnight at 4°C followed by appropriate HRP-conjugated secondary antibodies. Proteins were visualized using a Pierce enhanced chemiluminescence (ECL) kit (Invitrogen, USA) using the Odyssey Imaging System (LI-COR) and analyzed using Image Studio Lite v.5.2.5 (LI-COR).

#### Metabolic flux analysis

The real-time ATP production rate, and extracellular acidification rate (ECAR), and oxygen consumption rate (OCR) were measured using either an ATP Rate Assay or Mito Stress Test Kit, respectively, with an XF96 extracellular flux analyzer as per manufacturer’s instructions (Seahorse Biosciences). 400 000 BM neutrophils were seeded in assay medium (Agilent RPMI pH 7.4 supplemented with 10 mM of glucose, 2 mM of glutamine, and 1 mM of pyruvate) in an XF plate coated with 0.001% poly-D-lysine hydrobromide (Sigma-Aldrich). Cells were rested for 1 h at 37°C without CO_2_ before analysis. Measurements for OCR and ECAR were taken before and after the addition of oligomycin (1.5 μ M), FCCP (2.5 μ M), and rotenone (1 μ M)/antimycin A (1 μ M). Two independent experiments were performed with at least six mice per group and four technical replicates per biological sample. Results were acquired and analyzed by Wave 2.6.1.53 software (Agilent).

#### TEM imaging of neutrophils

Purified neutrophils for TEM were fixed in 2% paraformaldehyde and 2.5% glutaraldehyde in 0.1M sodium cacodylate buffer for 2 h at room temperature. Then, samples were washed in buffer, and postfixed in 1% osmium tetroxide in buffer for 90 min. Samples were dehydrated through a graded ethanol series (50%,70%, 90% and 100% ethanol for 20 min each) followed by two propylene oxide changes for 30 min. Samples were then embedded in Quetol-Spurr resin. Samples were polymerized overnight at 60°C and 70-nm thick slices were obtained using a Leica UC7 ultramicrotome. Finally, samples were stained with uranyl acetate and lead citrate, and images were acquired on a Hitachi HT7800 TEM operated at 120 kV using a EMSIS XAROSA CMOS camera (Nanoscale Biomedical Imaging Facility, The Hospital for Sick Children, Toronto, Canada). Quantifications of neutrophil size and cytoplasm/nucleus ratio was performed with the ImageJ software.

### Quantification and statistical analysis

Statistical analysis was done using Prism 9 software (GraphPad Software, San Diego, California USA). Data are represented as the mean ± SD or median. All statistical comparisons were evaluated with either parametric or non-parametric unpaired, two-tailed student’s t-test (for two groups) or one-way ANOVA (for multiple groups) followed by Tukey’s, Šidák’s or Dunn’s multiple comparisons test. Kaplan-Meier survival was analyzed by Mantel-Cox Log -rank test. For statistical comparison of more than two groups with multiple time points, two-way ANOVA followed by Bonferroni post-hoc tests were used. *p* < 0.05 was considered statistically significant. For IF and TEM quantification, individuals were blinded to experimental groups. All of the statistical details of experiments can be found in the figure legends.

## Supplemental information

Document S1. Figures S1–S9 and Tables S1–S4

Document S2. Article plus supplemental information
