# Supplementary material for: Mitochondrial perturbations in low-protein-diet-fed mice are associated with altered neutrophil development and effector functions
Source: Cell Rep. 2024 Jul 18;43(8):114493. doi: 10.1016/j.celrep.2024.114493 (PMC11372442; doi:10.1016/j.celrep.2024.114493)
Supplement: Document S1. Figures S1–S9 and Tables S1–S4 [file mmc1.pdf]

Cell Reports, Volume *43*

Supplemental information

Mitochondrial perturbations in low-protein-diet-fed mice are associated with altered neutrophil development and effector functions

Mehakpreet K. Thind, Emiliano Miraglia, Catriona Ling, Meraj A. Khan, Aida Glembocki, Celine Bourdon, YueYing ChenMi, Nades Palaniyar, Michael Glogauer, Robert H.J. Bandsma, and Amber Farooqui

Supplemental Information

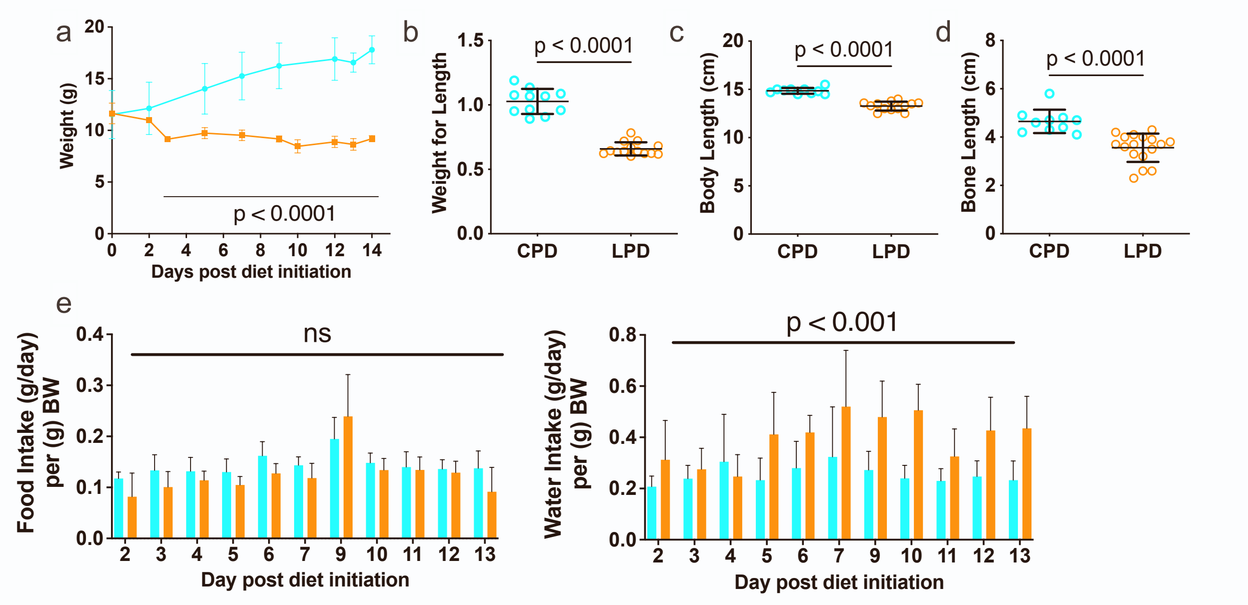

Figure S1: Reductions in anthropometric measurements in low-protein fed mice. CPD (cyan) and LPD (orange) protein fed; Mice fed low-protein diet show significant reductions in (a) Body weight assessed thrice weekly over the 14-day experimental period (n = 7/group) (b) Weight-for-length assessed on day 14 (n =10/group), (c-d) Body and bone length assessed on day 14 (n =10/group), and (e) food (*left)* & water *(right)* intake assessed throughout the 14-day experimental period. Results are expressed as means ± SD as determined by (a) two-way ANOVA (b-d) unpaired two-tailed t-test analysis (e) two-way ANOVA.

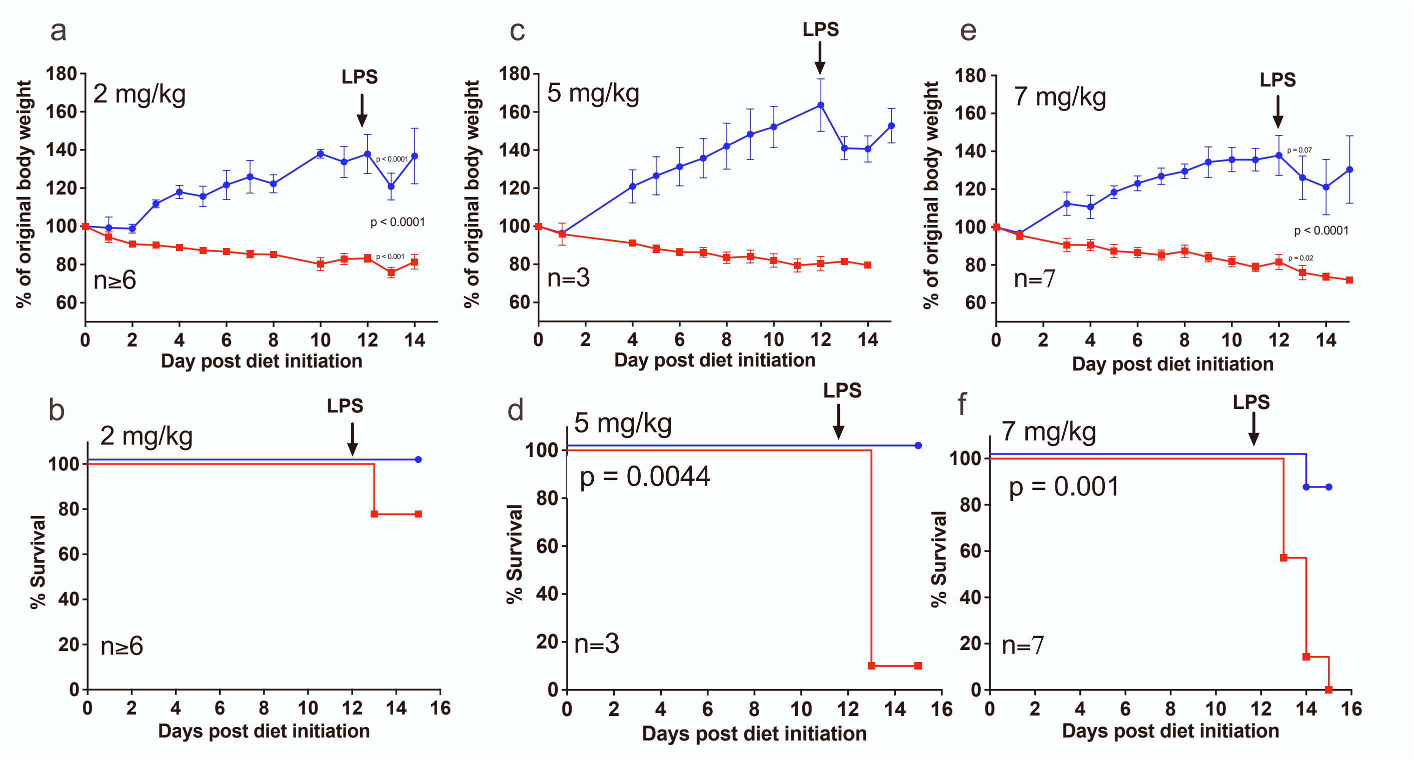

Figure S2: LPS (B55:05) dose-dependent response in mice fed a low-protein diet. Percent change of original body weight in CPD (blue) and LPD (red) fed mice with (a) 2 mg/kg (n=6) (c) 5 mg/kg (n=3) (e) 7 mg/kg (n=7) IP LPS on day 12 with follow-up till day 14. Percent survival was also determined in CPD (blue) and LPD (red) fed mice with (b) 2 mg/kg (n=6) (d) 5 mg/kg (n=3) (f) 7 mg/kg (n=7) LPS on day 12 with follow-up till day 14. Results are expressed as means ± SD as determined by (a,c,& e) unpaired two-tailed t-test analysis or (b,d, & f) two-sided long-rank (Mantel-Cox) test.

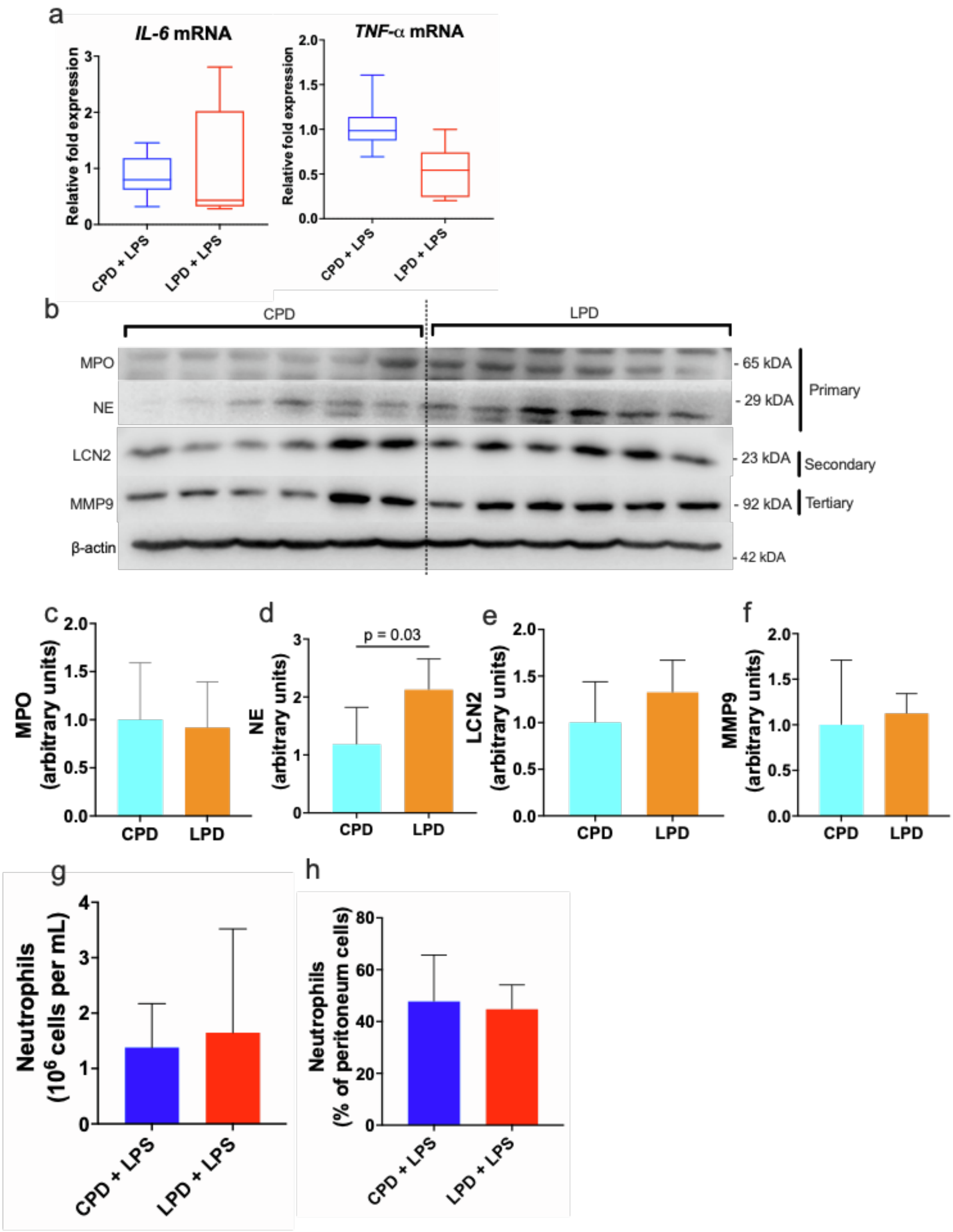

Figure S3: Low-protein diet alone is not associated with neutrophil accumulation. (a) Box-and-whisker plots of mRNA expression of cytokines (normalized to expression of Actinb in CPD + LPS; n=6/group). Results are expressed as median as determined by non-parametric Mann-Whitney test.

(b) Representative Western Blot probed for MPO, NE, LCN-2, and MMP9 and β-actin in lung lysates of CPD or LPD-fed mice is shown. Quantification of (c) MPO (d) NE (e) LCN-2, and (f) MMP9 normalized to the amount of β-actin was calculated (n=6/group). (g) The absolute number and (h) percentage of neutrophils (CD11b+Ly6G+ cells) in the peritoneal cavity. Results are expressed as means ± SD as determined by (c-h) unpaired two tailed t-test analysis.

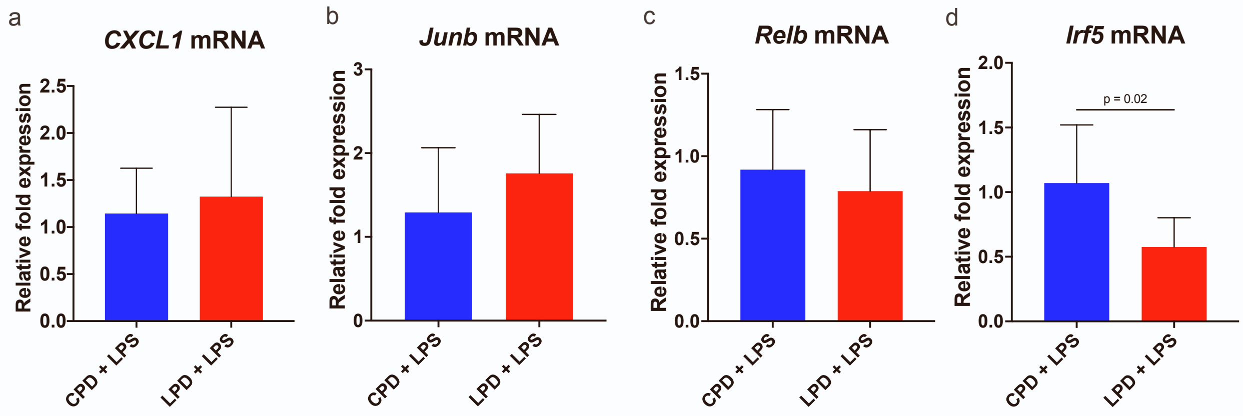

Figure S4: Neutrophil alterations are not associated with transcriptional rewiring at sites of inflammation. (a-d) qPCR analysis of gene expression (CXCL1, Junb, Relb, and Irf5; normalized to expression of Rpl13a in CPD + LPS) in lung lysates. Each sample was analyzed in triplicate wells. Results are expressed as means ± SD (ΔΔCt); n= 8/group. Significance is determined by unpaired two-tailed t-test analysis.

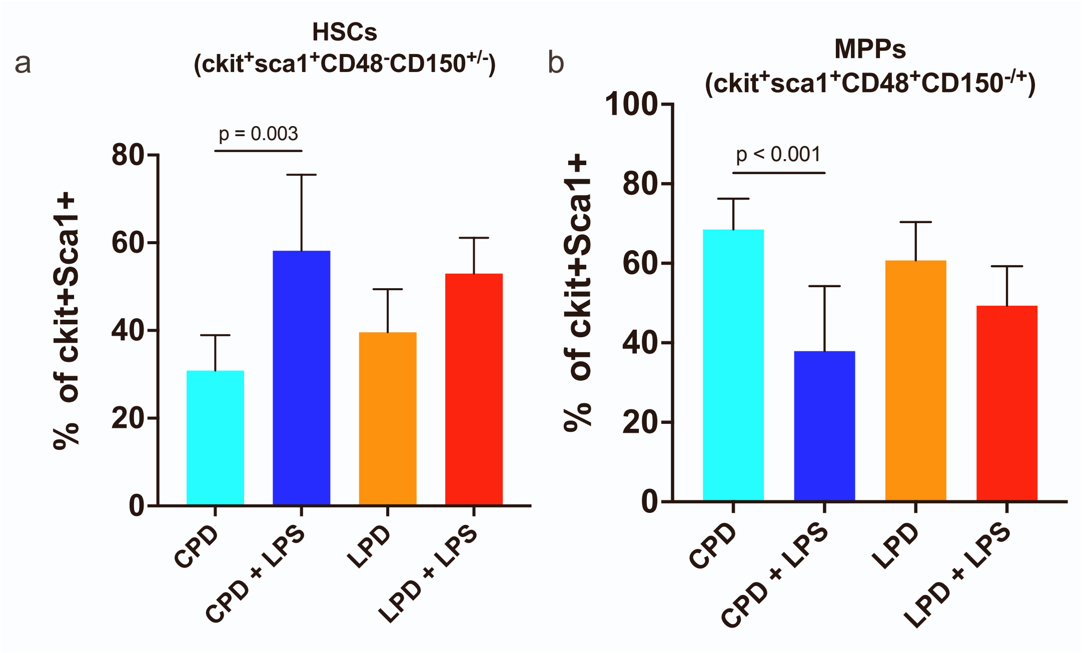

Figure S5: Early stem cells and progenitors are unaltered in protein malnutrition. Flow cytometry to compare the proportion of (a) HSCs (ckit^{+}sca1^{+}CD48^{-}CD150$^{+/-}$) and (b) MPPs (ckit^{+}sca1^{+}CD48^{+}CD150$^{-/+}$) in the BM. Results are expressed as means ± SD as determined by (a-b) one-way ANOVA with Tukey's multiple comparisons test (n=6/group).

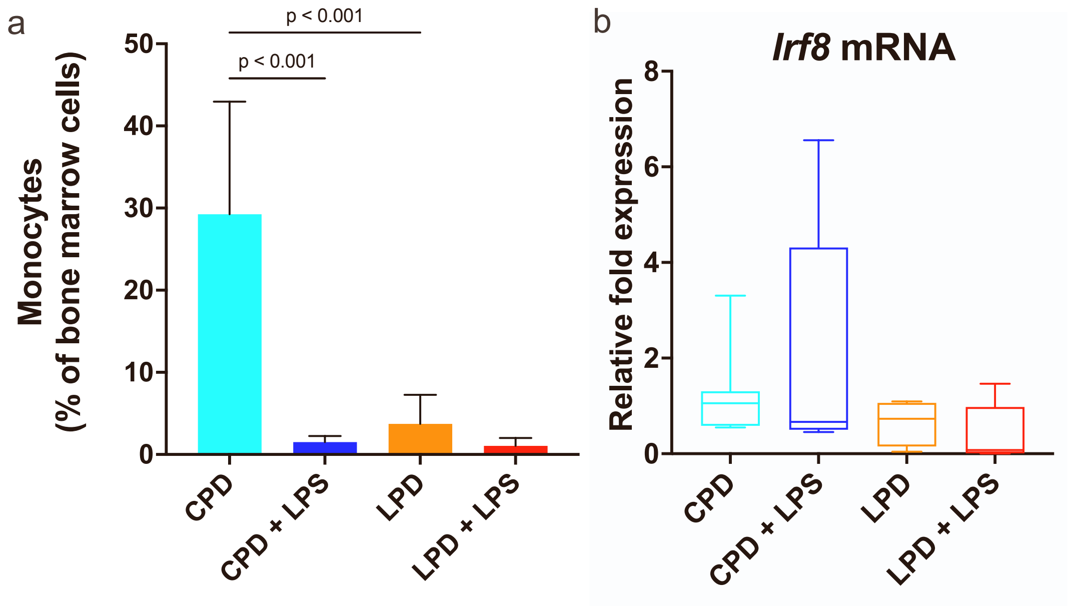

Figure S6: Low-protein diet does not affect monocyte differentiation. (a) Flow cytometry to compare (a) the proportion of monocytes (CD115+ cells) in the BM. (b) Box-and-whisker plots of the gene expression of Irf8 in CPD, CPD+LPS, LPD, and LPD + LPS diet (normalized to expression of Rpl13a in control mice without LPS). Each sample was analyzed in triplicate wells (n= 8/group). Results are expressed as (a) means ± SD as determined by one-way ANOVA or (b) median as determined by non-parametric Kruskal-Wallis test with multiple comparisons test.

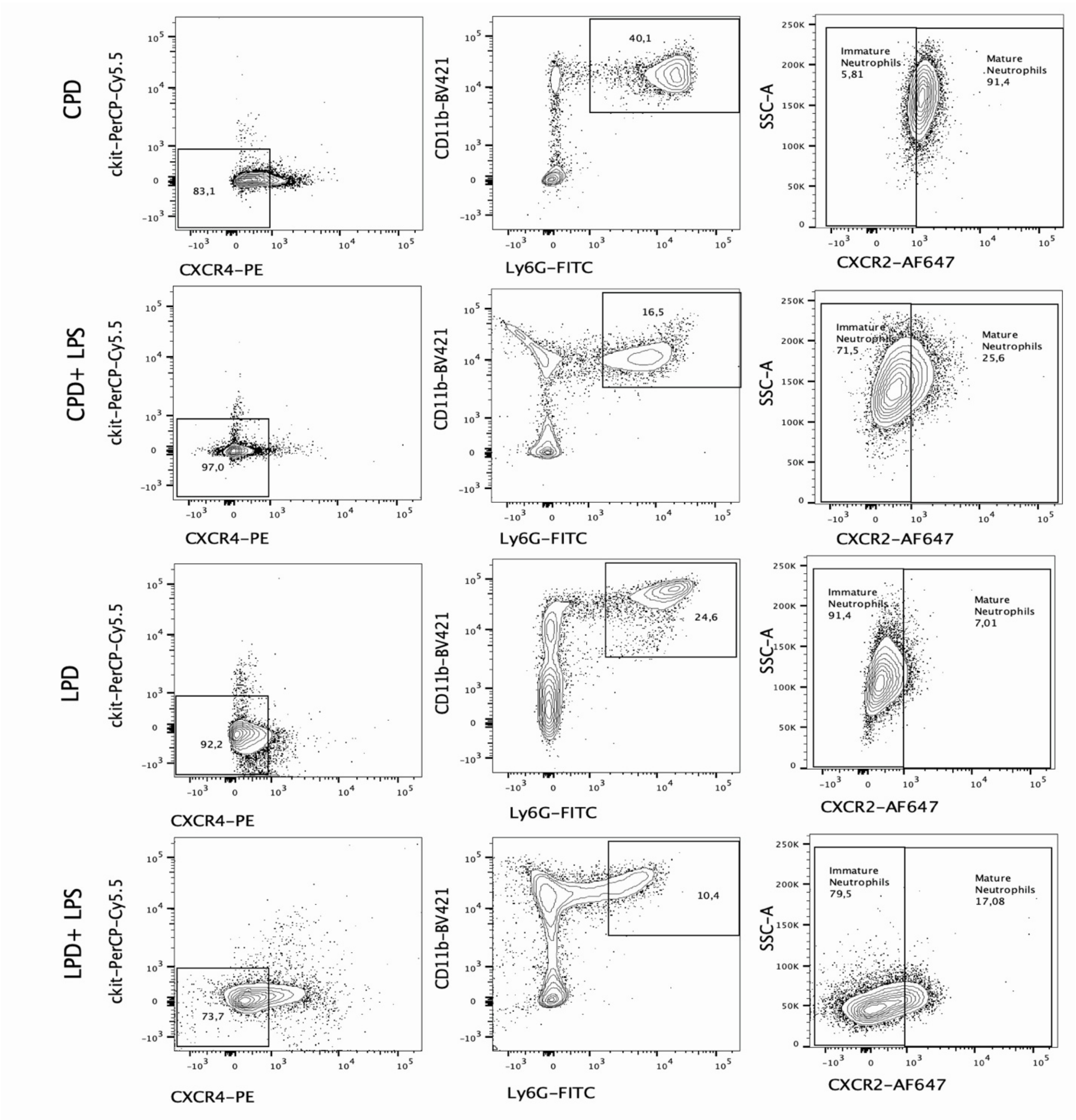

Figure S7: Representative gating strategy of bone marrow cells to identify immature and mature neutrophils. Numerical values represent the percentage of cells within each gate. Supplementary Table 5 provides a summary of this data.

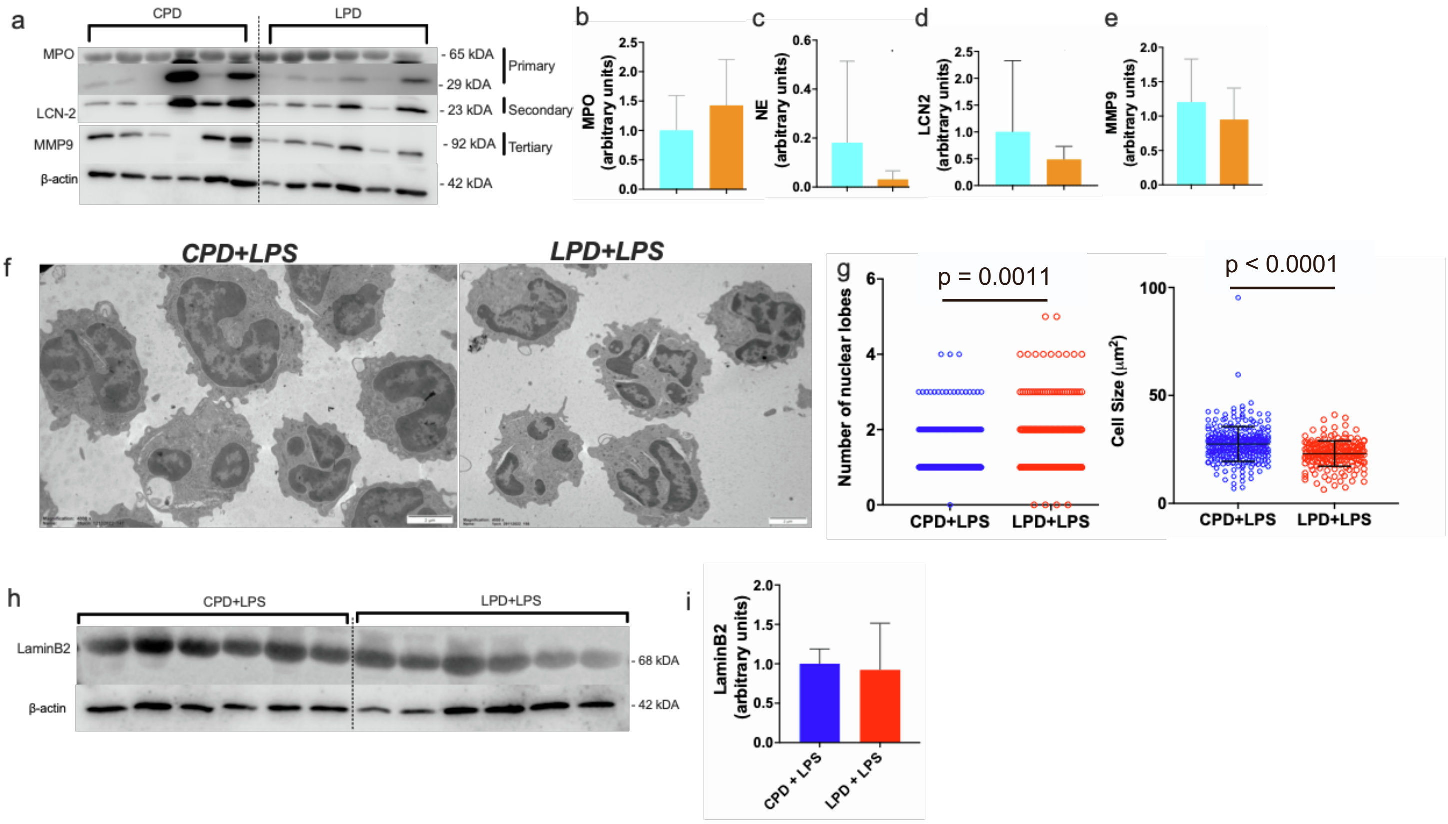

Figure S8: Morphological characterization of neutrophils. (a) Representative Western Blot probed for MPO, NE, LCN-2, and MMP9 and β-actin in BM neutrophils of CPD or LPD fed mice is shown. Quantification of (b) MPO (b) NE (d) LCN-2, and (e) MMP9 normalized to the amount of β-actin was calculated (n=6/group). (f) Representative images of electron micrographs to visualize BM neutrophils in LPS challenged CPD (*left*) and LPD (*right*) fed mice and (g; l*eft*), number of nuclear lobes and (g; *right*) cell size was quantified. At least 200 cells were counted from different fields and cells were pooled from 3 mice per group. Scale bar, 2 µm. (h) Representative Western Blot probed for LAMIN-B2 and β-actin in BM neutrophils of CPD+LPS or LPD+LPS fed mice is shown. Quantification of (i) LAMIN-B2 normalized to the amount of β-actin was calculated (n=6/group). Results are expressed as means ± SD as determined by (b-e; g; i) unpaired two tailed t-test analysis.

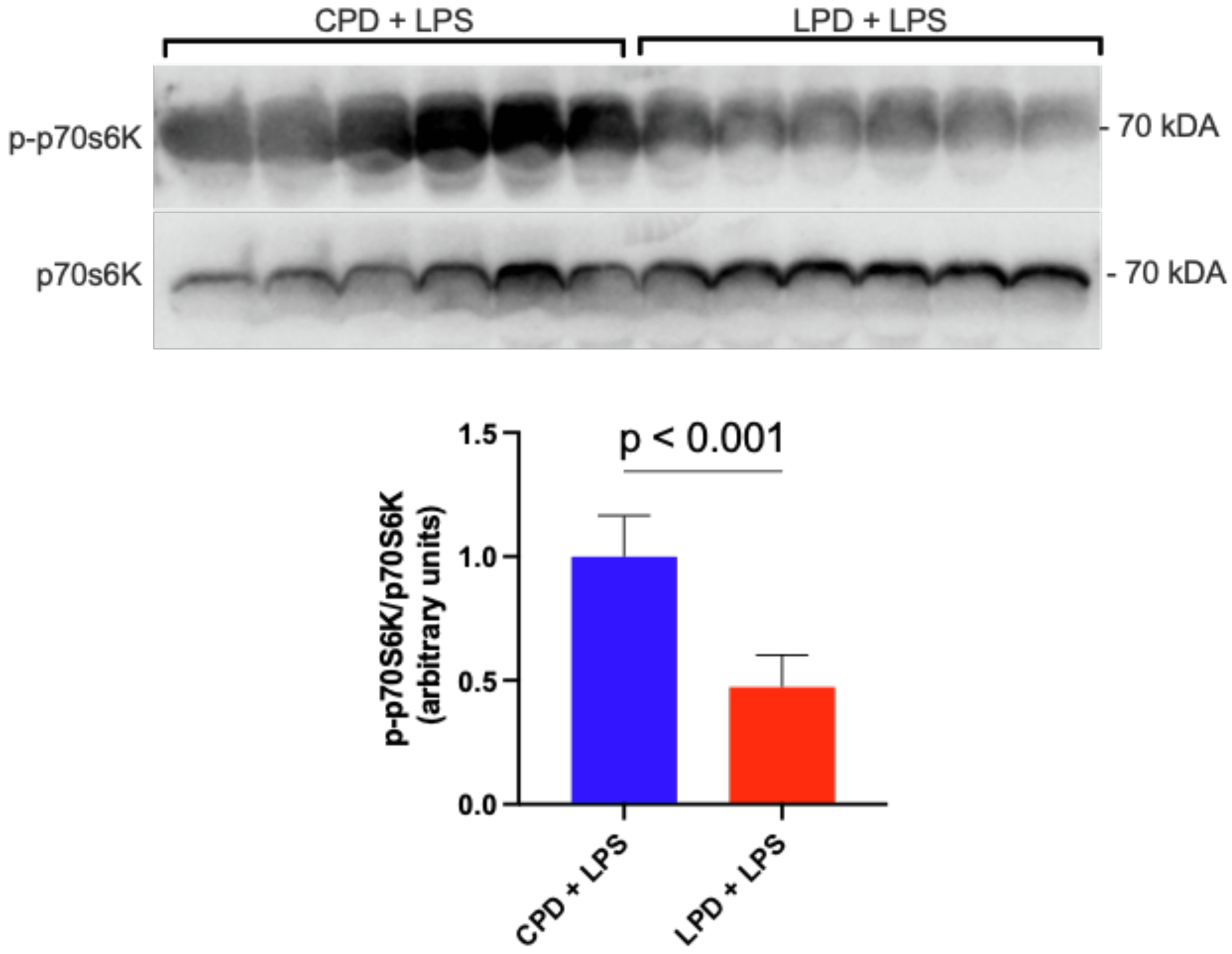

Figure S9: mTOR activity is suppressed in LPD neutrophils. *(Top)* Representative Western Blot probed for p-p70s6K p70s6K and β-actin in BM neutrophils of LPS challenged CPD or LPS fed mice is shown. *(Bottom)* Quantification normalized to the amount of total protein (n=6/group). Results are expressed as means ± SD as determined by unpaired two tailed t-test analysis.

Supplementary Table 1: Composition of the model diets

**Ingredient**	**18% Protein diet (g/Kg)**	**1% Protein Diet (g/Kg)**
Casein	207.0	11.5
DL- Methionine	2.7	0.36
Sucrose	350.0	350.0
Corn Starch	251.3	426.54
Maltodextrin	50.0	50.0
Corn Oil	52.6	54.2
Cellulose	41.06	60.6
Vitamin Mix	10.0	10.0
Ethoxyquin	0.01	0.01
Mineral Mix	13.37	13.37
Calcium Phosphate	17.36	22.32
Calcium Carbonate	4.6	1.1

Supplementary Table 2: Clinical Severity Scoring System to assess humane endpoints and minimize suffering to the animals

	**Clinical Evaluation**
Body Weight	20% BW decrease of beginning BW = **5**<br>20% decrease within 48h or 25% of begin BW = **35**
Dehydration	Normal = **0;** Dehydrated = **1**
Fur Quality	Normal = **0**<br>Ruffled = **5**<br>Rash; pallor; redness; icterus; wound; abscess; ulcer; petechiae; ecchymoses **= 10**
Respiration	Normal = **0**<br>Increased or decreased; Dyspnea (shortness of breath) = **5**<br>Open mouth breathing; cyanosis = **15**
Stool Consistency	Normal consistency; volume = **0**<br>Decreased feces; dry; wet and pasty; watery; discoloration = **2**<br>Feces absent = **5**<br>Liquid feces; blood in feces = **10**
Hunched Back	Normal = **0**<br>Hunched = **5**<br>Hunched; lethargic and no movement when touching = **10**
Movement	Normal = **0**<br>Slight hypoactivity = **2**<br>Hyperactivity; hypoactivity = **5**

	Ataxia (lack of voluntary coordination of muscle movements); neurologic signs tremors, head tilt) = **10**<br><br>Reluctant to move; uses cage for support; difficulty getting food or water; seizures= **15**<br><br>Down with no or minimal movement or response to human; coma = **35**
Grimace Scale	Normal = **0**<br><br>Score 1 = **5**<br><br>Score 2 = **10**

Supplementary Table 3: qPCR Primer Sequences

Target Gene	5'-Forward-3'	5'-Reverse-3'
Bmi1 (XM_036157771.1)	CGACGAGGTGGGTGTTAGG	AAACAACTGGGAACCGCCAT
Hoxa9 (NM_001277238.1)	CCGGACGGCAGTTGATAGAG	CTTCTTCCGAGTGGAGCGAG
Rag1 (NM_009019.2)	GACCCTTTGGGCATTGAGGA	ATGGCAATGTGCTAGGTGCT
Cebpa (Hsu et al., 2019)	AATGGCAGTGTGCACGTCTA	CCCCAGCCGTTAGTGAAGAG
G-CSFr (NM_001252651.1)	ATACCCCTCACCCACTACAC	ATGATACAAACTGGCGGGC
Cebpγ (NM_009884.3)	GAATAGTGACGAATACCGCC	GCTTTCTGCTTGCTTTTTAACC
Cebpe (Hsu et al., 2019)	GCAGCCACTTGAGTTCTCAGG	GATGTAGGCGGAGAGGTCGAT
Runx1 (NM_001111021.2)	CCGTCTTTACAAATCCGCC	GTCTGATCATCTAGTTTCTGCC
Klf6 (NM_011803.2)	GTTCGAAGTGGGACCTCTGG	AGCCATTAAAGTGGCACCGA
Cebpb (Hirai et al., 2006)	AAGCTGAGCGACGAGTACAAGA	GTCAGCTCCAGCACCTTGTG
Cebpδ	AGAACCCGCGGCCTTCTAC	ATGTAGGCGCTGAAGTCGAT

(Hsu et al., 2019)		
Cebpζ<br><br>(Hsu et al., 2019)	ACAGGGGTGAACAGAGCATAC	AGCATTAAAGCCTGCACACTC
IL-1beta<br><br>(NM_008361.4)	TTCCCCAGGGCATGTTAAGG	CTTGGCCGAGGACTAAGGAG
IL-10<br><br>(NM_010548.2)	GCTCTTGCACTACCAAAGCC	CTGCTGATCCTCATGCCAGT
TNFa<br><br>(NM_013693.3)	AGTACTTAGACTTTGCGGAG	CTGGGTAGAGAATGGATGAAC
IL-6<br><br>(NM_031168.2)	GTGGCTAAGGACCAAGACCA	GGTTTGCCGAGTAGACCTCA
ActinB<br><br>(NM_007393.5)	GCAGGAGTACGATGAGTCCG	ACGCAGCTCAGTAACAGTCC
Rpl13a<br><br>(NM_009438.5)	TCCCTCCACCCTATGACAAG	GTCACTGCCTGGTACTTCC
mtDNA	CCCAGCTACTACCATCATTCAAGT	GATGGTTTGGGAGATTGGTTGATGT
Beta globin	AAGGTGAACGCCGATGAAGT	ATCAAAGTACCGCTGGGTCC

Supplementary Table 4: Summary Data of Bone Marrow Neutrophils

	Ly6G+ Neus Mean ±SD (95% CI)	Immature Neus Mean ±SD (95% CI)
CPD	31.70±2.17 (29.70-33.71)	1.22±0.62 (0.57-1.88)
CPD + LPS	12.09±3.44 (9.78-14.41)	7.42±2.51 (5.83-9.02)
LPD	38.40±3.95 (35.10-41.71)	25.57±4.01 (21.86-29.28)
LPD + LPS	11.20±3.99 (8.52-13.87)	11.41±4.68 (8.26-14.55)
